# Advancing Breast Cancer Treatment: The Role of Immunotherapy and Cancer Vaccines in Overcoming Therapeutic Challenges

**DOI:** 10.3390/vaccines13040344

**Published:** 2025-03-24

**Authors:** Marco Palma

**Affiliations:** Institute for Globally Distributed Open Research and Education (IGDORE), 03181 Torrevieja, Spain; marco.palma@igdore.org

**Keywords:** breast cancer, immunotherapy, cancer vaccines, HER2-positive, triple-negative breast cancer, tumor microenvironment

## Abstract

Breast cancer (BC) remains a significant global health challenge due to its complex biology, which complicates both diagnosis and treatment. Immunotherapy and cancer vaccines have emerged as promising alternatives, harnessing the body’s immune system to precisely target and eliminate cancer cells. However, several key factors influence the selection and effectiveness of these therapies, including BC subtype, tumor mutational burden (TMB), tumor-infiltrating lymphocytes (TILs), PD-L1 expression, HER2 resistance, and the tumor microenvironment (TME). BC subtypes play a critical role in shaping treatment responses. Triple-negative breast cancer (TNBC) exhibits the highest sensitivity to immunotherapy, while HER2-positive and hormone receptor-positive (HR+) subtypes often require combination strategies for optimal outcomes. High TMB enhances immune responses by generating neoantigens, making tumors more susceptible to immune checkpoint inhibitors (ICIs); whereas, low TMB may indicate resistance. Similarly, elevated TIL levels are associated with better immunotherapy efficacy, while PD-L1 expression serves as a key predictor of checkpoint inhibitor success. Meanwhile, HER2 resistance and an immunosuppressive TME contribute to immune evasion, highlighting the need for multi-faceted treatment approaches. Current breast cancer immunotherapies encompass a range of targeted treatments. HER2-directed therapies, such as trastuzumab and pertuzumab, block HER2 dimerization and enhance antibody-dependent cellular cytotoxicity (ADCC), while small-molecule inhibitors, like lapatinib and tucatinib, suppress HER2 signaling to curb tumor growth. Antibody–drug conjugates (ADCs) improve tumor targeting by coupling monoclonal antibodies with cytotoxic agents, minimizing off-target effects. Meanwhile, ICIs, including pembrolizumab, restore T-cell function, and CAR-macrophage (CAR-M) therapy leverages macrophages to reshape the TME and overcome immunotherapy resistance. While immunotherapy, particularly in TNBC, has demonstrated promise by eliciting durable immune responses, its efficacy varies across subtypes. Challenges such as immune-related adverse events, resistance mechanisms, high costs, and delayed responses remain barriers to widespread success. Breast cancer vaccines—including protein-based, whole-cell, mRNA, dendritic cell, and epitope-based vaccines—aim to stimulate tumor-specific immunity. Though clinical success has been limited, ongoing research is refining vaccine formulations, integrating combination therapies, and identifying biomarkers for improved patient stratification. Future advancements in BC treatment will depend on optimizing immunotherapy through biomarker-driven approaches, addressing tumor heterogeneity, and developing innovative combination therapies to overcome resistance. By leveraging these strategies, researchers aim to enhance treatment efficacy and ultimately improve patient outcomes.

## 1. Introduction

Breast cancer (BC) remains a significant global health challenge, with its complex biology presenting both diagnostic and therapeutic hurdles. Surgery is the cornerstone of BC treatment, often combined with other modalities, such as radiation, chemotherapy, hormone therapy, or neoadjuvant therapies. The treatment approach is generally tailored to the cancer’s stage, molecular subtype, and the individual patient’s characteristics. For early-stage BC, breast-conserving surgery (BCS) is considered the gold standard, typically followed by adjuvant radiotherapy to reduce the risk of local recurrence. BCS aims to remove the tumor, while preserving as much healthy tissue as possible, striving to balance oncologic safety with acceptable cosmetic outcomes [1].

For estrogen receptor (ER)-positive BCs, hormone therapy remains a cornerstone of treatment, often utilized alongside surgery. Agents such as Tamoxifen and gonadotropin-releasing hormone agonists (GnRHa) have long been the mainstay of treatment for ER-positive cancers [2]. More recently, advances in endocrine therapy, including the introduction of aromatase inhibitors and fulvestrant, have provided additional therapeutic options and have significantly improved patient outcomes [3]. These therapies have shown promise in reducing the risk of recurrence, offering patients with ER-positive BC more favorable long-term prognoses.

However, despite these advancements, there are inherent challenges in the management of BC. Recurrence, drug resistance, and the long-term side effects of conventional treatments remain pressing concerns, particularly in more aggressive forms of BC. These issues highlight the critical need for novel treatment strategies that are more targeted and less toxic. In this regard, immunotherapy has emerged as a promising alternative, especially for challenging subtypes, such as triple-negative breast cancer (TNBC) [4]. Immunotherapy harnesses the body’s immune system to specifically target and eliminate cancer cells, offering the potential for more effective and less harmful treatment options compared to traditional therapies.

One of the most exciting and emerging concepts in BC immunotherapy is the use of cancer vaccines, which aim to stimulate a patient’s immune system to recognize and attack cancer cells. However, the success of immunotherapy, including cancer vaccines, is highly dependent on the patient’s immune system’s ability to mount an effective response. Several factors, including cancer’s intrinsic immunogenicity and the patient’s immune capacity, play crucial roles in determining whether immunotherapy will be successful. These factors underscore the importance of personalized treatment strategies that consider both the immune response capabilities of the patient and the unique characteristics of their cancer.

In this review, we will explore key factors that influence the success of immune response-based therapies for BC. Topics such as BC classification, strategies for anticancer immunotherapy, and the importance of assessing the immunogenicity of tumors before initiating treatment will be discussed. Additionally, we will focus on the potential of anticancer vaccines in BC, with particular emphasis on epitope-based vaccines, which represent a cutting-edge approach to targeting specific tumor-associated antigens. By identifying and understanding the crucial factors that influence the effectiveness of immunotherapy, this review aims to provide insights into how personalized immunotherapy could lead to more effective and tailored treatment options for BC patients.

## 2. Key Factors Influencing Immunotherapy and Vaccine Selection in Breast Cancer

There are some important factors in breast cancer that may affect the decision of which immunotherapy or type of vaccine should be applied to obtain a better outcome. Some of these factors are types of breast cancer, tumor mutation burden (TMB), tumor-infiltrating lymphocytes (TILs), PDL-1 expression, HER2 resistance, and tumor microenvironment (TME) (Table 1). The distribution of these factors across various breast cancer subtypes has been analyzed in multiple studies (Table 2).

### 2.1. Breast Cancer Classification

Breast cancer classification is essential for prognosis and treatment decisions, based on tissue origin, molecular characteristics, and receptor status.

Breast cancer is categorized as non-invasive or invasive [5]. Non-invasive cancer, or carcinoma in situ, remains confined to ducts or lobules without spreading. Ductal carcinoma in situ (DCIS) is a precursor to invasive ductal carcinoma (IDC) and is often detected via mammography [16]. Lobular carcinoma in situ (LCIS) increases future breast cancer risk but does not typically progress to invasive cancer [17]. The early detection of non-invasive cancers results in excellent prognosis, with treatment involving surgery and, in some cases, radiation or hormone therapy.

Invasive breast cancer extends beyond ducts or lobules, potentially metastasizing to lymph nodes or distant organs. The most common subtypes are IDC, accounting for 72–80% of cases [18], and invasive lobular carcinoma (ILC), constituting 5–15% [18]. IDC originates in milk ducts and spreads to adjacent tissues, often presenting as a palpable lump. ILC, characterized by E-cadherin protein loss, spreads in a diffuse pattern, making detection difficult. Aggressive subtypes, such as inflammatory breast cancer and triple-negative breast cancer (TNBC), present treatment challenges due to rapid progression and therapy resistance. Treatment for invasive cancers includes surgery, radiation, chemotherapy, targeted therapy, and endocrine therapy.

Molecular and receptor status significantly impact prognosis and treatment. Classification is based on estrogen receptor (ER), progesterone receptor (PR), and human epidermal growth factor receptor 2 (HER2) expression. The four intrinsic subtypes are luminal A, luminal B, HER2-enriched, and TNBC [19].

Luminal A (50–60% of cases) [19] is ER+ and/or PR+ with low Ki-67 levels, indicating slow proliferation [20]. It has a favorable prognosis and is treated with hormone therapy, surgery, and radiation. Luminal B (10–20%) exhibits higher Ki-67 levels and, in some cases, HER2 positivity, making it more aggressive. Treatment includes endocrine therapy, chemotherapy, and HER2-targeted therapies. HER2-enriched breast cancer (15–20%) [19] lacks hormone receptor expression but overexpresses HER2, leading to rapid growth. Targeted therapies, such as trastuzumab and pertuzumab, have improved survival. TNBC (10–15%) lacks ER, PR, and HER2 expression [21], making hormone and HER2-targeted therapies ineffective. It is associated with high Ki-67 expression and early metastasis, requiring chemotherapy as the primary treatment. Emerging treatments, such as immune checkpoint and PARP inhibitors, offer new hope.

Breast cancer staging ranges from stage 0 (in situ cancers) to stage IV (metastatic disease). The complexity of classification highlights the need for individualized treatment. Advances in molecular profiling and targeted therapies continue to improve patient outcomes by providing more precise and effective strategies.

### 2.2. Tumor Mutation Burden (TMB)

TMB represents the total number of mutations per coding region of a tumor genome. It is commonly measured in mutations per megabase (mut/Mb) and reflects the prevalence of non-synonymous somatic mutations in tumor DNA. TMB has emerged as a promising biomarker for predicting the likelihood of response to ICIs and other immunotherapies [22].

TMB is determined using the next-generation sequencing (NGS) of tumor samples [23]. While methodologies can vary across sequencing panels, comprehensive genomic profiling is often employed to ensure accurate assessment. Tumors with a high TMB tend to produce more neoantigens [6], which are novel peptides recognizable by the immune system. As a result, such tumors are potentially more responsive to immunotherapy. High TMB levels have been correlated with improved outcomes in cancers treated with ICIs, such as PD-1/PD-L1 or CTLA-4 inhibitors [24].

In breast cancer, TMB is generally lower compared to malignancies like melanoma or non-small cell lung cancer [23]. However, specific subtypes, such as TNBC, may exhibit higher TMB in certain cases, making them potential candidates for immunotherapy [25]. While clinical trials have begun exploring the relationship between TMB and immunotherapy efficacy in BC, this area remains under active investigation.

A threshold of TMB, often ≥10 mut/Mb, is commonly used to classify tumors as “TMB-high” in clinical studies [26]. However, this cutoff can vary based on the type of cancer and the assay used for measurement.

It is important to note that TMB is only one of several factors influencing response to immunotherapy. Other biomarkers, including PD-L1 expression, immune cell infiltration, and the characteristics of the TME, play significant roles. The use of TMB as a predictive biomarker in BC is less established than in other cancer types, and its predictive power may depend on the specific context.

### 2.3. Tumour-Infiltrating Lymphocytes (TILs)

TME critically influences the antitumor immune response. High levels of CD8+ TILs are generally associated with improved clinical outcomes [7]; whereas, an abundance of regulatory T-cells (Tregs) can contribute to immune suppression and tumor progression [27]. Breast cancer, traditionally considered immunologically “cold” due to its relatively low mutation burden compared to other cancers, still demonstrates heterogeneity in immune infiltration. TNBC, for example, tends to have higher TIL levels compared to hormone receptor-positive (HR+) and HER2-positive subtypes, highlighting distinct immune microenvironments among breast cancer subtypes.

The presence and density of TILs are increasingly recognized as prognostic indicators in breast cancer. In TNBC, high TIL levels correlate with better survival outcomes and a reduced risk of recurrence, particularly when treated with chemotherapy [28]. Similarly, in HER2-positive BC, elevated TILs have been associated with improved responses to anti-HER2 targeted therapies, such as trastuzumab and pertuzumab [29]. TILs are also predictive biomarkers for the efficacy of immunotherapies, including ICIs. PD-1 and its ligand PD-L1 play central roles in immune evasion by tumors, and ICIs targeting these molecules have shown promising results in BC, particularly in TNBC. Patients with higher TIL levels and PD-L1 expression tend to derive greater benefit from these therapies [30].

Harnessing TILs for immunotherapy in breast cancer involves both endogenous and adoptive cell transfer (ACT) strategies. Adoptive TIL therapy entails isolating TILs from patient tumor biopsies, expanding them ex vivo, and reinfusing the activated T-cells into the patient [31]. This personalized approach aims to enhance antitumor immunity and has demonstrated encouraging results in early clinical trials [32], particularly in heavily pretreated TNBC patients [33]. The integration of TIL therapy with ICIs, chemotherapy, or targeted therapies represents a promising avenue to overcome immune suppression and enhance therapeutic efficacy. Preclinical and clinical studies suggest that combinatorial approaches can synergistically boost TIL activity [34], resulting in improved tumor control.

Despite their potential, the clinical implementation of TIL-based therapies in breast cancer faces several challenges, including the immunosuppressive TME, heterogeneity in TIL composition, and variability in patient responses [35]. Further research is needed to identify biomarkers that can predict responses to TIL therapies and optimize patient selection [36]. Future directions include enhancing TIL persistence and functionality through genetic engineering, such as chimeric antigen receptor (CAR)-TILs [37] or cytokine-based activation strategies [38]. Additionally, efforts to modulate the TME to favor TIL recruitment and activation, such as combination therapies with immune modulators or epigenetic drugs, hold promise for improving outcomes.

TILs represent a critical component of the antitumor immune response in breast cancer, with significant implications for prognosis and therapy. Advances in TIL-based immunotherapy, combined with emerging strategies to enhance TIL function and overcome immune resistance, offer new hope for patients with breast cancer, particularly in aggressive subtypes, like TNBC. Continued research into the biology and therapeutic potential of TILs will be essential to fully realize their role in breast cancer immunotherapy.

### 2.4. PD-L1 Expression

Knowing the PD-L1 expression in BC prior to immunotherapy is essential for optimizing treatment outcomes, as it significantly influences decision making and therapy effectiveness [8,39]. PD-L1 is widely recognized as a biomarker used to predict the likelihood of response to ICIs, such as anti-PD-1 or anti-PD-L1 antibodies. This is particularly important in breast cancer, especially in TNBC, which exhibits varying levels of PD-L1 expression. Testing for PD-L1 helps identify patients who are most likely to benefit from immunotherapy, as those with higher levels of expression generally show better responses to these treatments. ICIs work by blocking the PD-1/PD-L1 interaction, thereby reactivating T-cells to attack the tumor. Conversely, patients with low or absent PD-L1 expression may not benefit as significantly and might require alternative strategies. PD-L1 testing also supports a personalized treatment approach, ensuring tailored plans that avoid unnecessary exposure to ineffective therapies, while guiding combination therapies, such as immunotherapy with chemotherapy, for patients with low or heterogeneous PD-L1 expression. Additionally, PD-L1 levels hold prognostic value, as high expression can correlate with a more aggressive tumor phenotype yet predict a better response to immunotherapy. Regulatory and reimbursement considerations further emphasize the importance of PD-L1 testing, as many agencies and insurance providers mandate it to approve immunotherapy, ensuring cost-effective and evidence-based care. However, challenges such as tumor heterogeneity and variability in testing methods can complicate the process, highlighting the need for standardized approaches. Ultimately, PD-L1 testing plays a pivotal role in advancing precision medicine, ensuring that the right patients receive the most effective treatments for BC.

### 2.5. HER2 Resistance

HER2-positive breast cancer accounts for approximately 15–20% of all breast cancers [40] and is associated with an aggressive clinical course [41]. While anti-HER2 therapies, such as trastuzumab and pertuzumab, have significantly improved survival rates, some patients develop or exhibit primary resistance, reducing the efficacy of these treatments.

Identifying HER2 resistance prior to initiating immunotherapy is essential for optimizing treatment strategies and improving patient outcomes. Understanding resistance status enables clinicians to select therapies that specifically target immunosuppressive factors, thereby enhancing the effectiveness of immunotherapy. Moreover, HER2 resistance serves as a critical prognostic indicator, as it is associated with poorer outcomes and diminished responsiveness to standard anti-HER2 treatments. Comprehensive testing for resistance provides invaluable insights, allowing for timely and tailored therapeutic decisions that maximize the likelihood of success, while minimizing delays.

HER2 resistance plays a pivotal role in reshaping the TME, often suppressing the immune system’s ability to mount an effective antitumor response. This resistance is frequently associated with immunosuppressive changes, including an increase in regulatory T-cells (Tregs) [42] and myeloid-derived suppressor cells (MDSCs). Additionally, the mechanisms driving resistance to HER2 inhibitors are diverse and vary across HER2-positive breast cancer subtypes [43]. Cancer cells also employ a range of strategies to evade anti-HER2 therapies [44], further complicating treatment. Understanding and addressing these resistance mechanisms is critical for improving therapeutic outcomes.

Resistance to HER2-targeted therapies can significantly alter the immune landscape of tumors, often reducing immune cell infiltration or increasing immunosuppressive factors. This highlights the need for tailored immunotherapy approaches. Combining ICIs, such as anti-PD-1/PD-L1, with anti-HER2 agents has shown promise in mitigating these effects and improving therapeutic outcomes [9].

Testing for HER2 resistance is essential for identifying patients who may benefit from innovative immunotherapy strategies [45,46], including vaccines, adoptive T-cell therapies, or antibody-drug conjugates, like trastuzumab deruxtecan. These patients often require integrated treatment approaches that combine immunotherapy with targeted therapies, chemotherapy, or other agents to overcome resistance mechanisms and enable a more effective and personalized treatment strategy.

Moreover, identifying HER2-resistant patients prior to treatment allows for their enrollment in clinical trials investigating novel immunotherapy combinations or next-generation HER2-targeted therapies. This approach provides access to cutting-edge treatments that can significantly improve prognosis. However, challenges persist in HER2 resistance testing, such as the heterogeneity of HER2 expression and resistance mechanisms within tumors and across metastatic sites [47]. Complex underlying mechanisms, including HER2 gene mutations [48] or the activation of alternative signaling pathways [49], necessitate advanced diagnostic tools for precise detection.

In summary, testing for HER2 resistance is a critical component of precision oncology. It enables tailored treatment strategies, maximizes therapeutic efficacy, and guides the integration of immunotherapy with other modalities to overcome resistance. By understanding the mechanisms of driving resistance and their effects on the TME, clinicians can develop more effective approaches to improve outcomes for patients with HER2-positive breast cancer.

### 2.6. Tumor Microenvironment (TME)

The TME consists of cancer cells, stromal components, blood vessels, and infiltrating immune cells, all of which interact dynamically to influence tumor progression and therapeutic response [10].

#### 2.6.1. Cancer Cells: Heterogeneity and Adaptability

Breast cancer cells exhibit substantial heterogeneity, both within the same tumor (intratumoral heterogeneity) and among different patients (intertumoral heterogeneity) [50]. This heterogeneity arises from genetic mutations, epigenetic modifications, and interactions with the surrounding TME. Cancer cells can dynamically adapt to environmental stressors, such as hypoxia [51], metabolic shifts [52], and immune surveillance [53], facilitating their survival and resistance to therapies. TNBC cells, for example, are particularly aggressive, relying on immune suppression and stromal interactions to thrive [54].

#### 2.6.2. Stromal Cells: The Supportive Niche

The stromal compartment of the breast TME consists of fibroblasts, adipocytes, and extracellular matrix (ECM) components, all of which contribute to tumor progression by supporting cancer cell survival, invasion, and immune evasion [55,56].

Cancer-associated fibroblasts (CAFs) play a pivotal role by secreting growth factors such as TGF-β [57], VEGF [58], and IL-6 [59], which drive tumor cell proliferation, invasion, and resistance to apoptosis, while also remodeling the ECM to create a stiffened tumor bed that enhances cancer cell migration and immune suppression.

Adipocytes, abundant in breast tissue, significantly influence tumor growth by supplying free fatty acids [60], cytokines [61], like leptin [62] and IL-8 [63], and pro-inflammatory signals that fuel tumor metabolism and invasion.

The ECM, far from being merely a structural component, actively regulates cell signaling and is frequently modified by tumor cells to establish an immunosuppressive barrier, thereby restricting immune cell infiltration and promoting angiogenesis, ultimately facilitating tumor progression [64].

#### 2.6.3. Vasculature: Tumor Angiogenesis and Hypoxia

Breast tumors depend on abnormal angiogenesis to sustain rapid growth, but the resulting vasculature is often disorganized and leaky, leading to hypoxic regions that drive cancer aggressiveness and therapy resistance. Hypoxia-inducible factors (HIFs), particularly HIF-1α, are upregulated in response to low oxygen levels, promoting VEGF secretion and the formation of aberrant blood vessels that inefficiently deliver nutrients and immune cells [65]. This dysfunction not only fuels tumor progression but also creates a hostile microenvironment that impairs immune infiltration. Additionally, tumor endothelial cells actively interact with immune cells, influencing extravasation and immune exclusion. By exploiting these interactions, tumors can evade immune surveillance through the expression of PD-L1 [66] and other immunosuppressive molecules on endothelial surfaces, further enhancing their ability to resist immune-mediated destruction.

#### 2.6.4. Infiltrating Immune Cells: A Double-Edged Sword

The immune landscape of the breast TME is highly heterogeneous, consisting of both pro-tumorigenic and antitumorigenic immune cells that influence disease progression and treatment response. TAMs are often polarized toward an M2 phenotype, secreting IL-10, TGF-β, and VEGF, which foster immune suppression and angiogenesis, with high TAM infiltration correlating with poor prognosis in aggressive breast cancers, like TNBC [67]. Regulatory T-cells (Tregs) further contribute to immune evasion by secreting IL-10 and TGF-β, suppressing cytotoxic T-cell responses, and creating an immunosuppressive microenvironment that shields tumors from immune attack [68]. Similarly, myeloid-derived suppressor cells (MDSCs) inhibit T-cell activation and promote resistance to immunotherapy [69], presenting a major challenge in breast cancer treatment. While cytotoxic T lymphocytes (CTLs) have the potential to eliminate cancer cells, their function is often suppressed due to the upregulation of immune checkpoint molecules, such as PD-1 and CTLA-4, a barrier that immunotherapies aim to overcome. Natural killer (NK) cells also play a critical role in tumor cell recognition and destruction; however, breast tumors evade NK-mediated cytotoxicity by downregulating MHC class I molecules and secreting immunosuppressive factors [70]. This intricate balance between immune activation and suppression within the TME underscores the need for targeted immunotherapeutic strategies to enhance antitumor immunity.

#### 2.6.5. Crosstalk Between TME Components

Breast cancer progression is driven by complex crosstalk between these TME components [71]. For instance, CAFs enhance immune evasion by recruiting Tregs and MDSCs, while TAMs promote angiogenesis, reinforcing hypoxia-driven tumor aggressiveness. Additionally, exosomes and extracellular vesicles (EVs) secreted by cancer and stromal cells carry oncogenic signals, further shaping the microenvironment.

#### 2.6.6. Therapeutic Implications: Targeting the Breast TME

Given the crucial role of the TME in breast cancer progression, emerging therapies aim to reprogram its components to enhance treatment efficacy [72]. Immune checkpoint inhibitors, such as anti-PD-1/PD-L1 and anti-CTLA-4 therapies, seek to restore T-cell function, though their effectiveness in breast cancer, particularly TNBC, remains under investigation. Strategies targeting TAMs, such as CSF1R inhibition, aim to reduce immunosuppressive macrophages and reinvigorate antitumor immunity [73]. Anti-angiogenic therapies, including VEGF inhibitors like sunitinib and bevacizumab, help normalize the tumor vasculature, improving immune cell infiltration and drug delivery [74]. Cancer vaccines targeting TAAs within the TME are being explored to stimulate tumor-specific immune responses [75]. Additionally, TME-targeted nanoparticles represent a promising nanomedicine approach, enabling the selective delivery of therapeutic agents to specific TME components, such as fibroblasts or immunosuppressive cells, to enhance treatment outcomes. These targeted strategies reflect the growing recognition that effective breast cancer therapies must address not only tumor cells but also the supportive and suppressive elements of the TME.

#### 2.6.7. Conclusions

The breast tumor microenvironment plays a critical role in shaping cancer progression, immune evasion, and therapeutic resistance. A deeper understanding of the dynamic interactions between cancer cells, stromal elements, vasculature, and immune infiltrates can inform the design of next-generation immunotherapies and targeted treatments. Future strategies should focus on overcoming TME-mediated immune suppression to enhance antitumor immunity and improve patient outcomes.

## 3. Anticancer Immunotherapy Strategy Against Breast Cancer

Breast cancer remains one of the most prevalent and challenging malignancies worldwide [76], necessitating the development of innovative therapeutic approaches. Immunotherapy has emerged as a promising strategy, leveraging the body’s immune system to target and eliminate cancer cells [77]. Unlike conventional treatments, such as chemotherapy and radiation, immunotherapy aims to enhance the immune system’s ability to recognize and attack tumor cells with greater specificity, minimizing damage to healthy tissues [78]. Recent advancements, including ICIs, cancer vaccines, and adoptive T-cell therapy, have shown significant potential in improving outcomes for breast cancer patients. This section explores the various immunotherapeutic strategies designed to combat breast cancer, their mechanisms of action, and the challenges and opportunities they present in clinical applications (Table 3).

### 3.1. HER2 Block: Targeting Oncogenic Signaling Pathways in Cancer Therapy

HER2 (human epidermal growth factor receptor 2), also known as ERBB2, is a transmembrane glycoprotein and tyrosine kinase receptor that belongs to the ERBB family of epidermal growth factor receptors (EGFRs). It is a key regulator of intracellular signaling pathways that govern essential cellular processes, including cell growth, proliferation, survival, differentiation, and migration [85]. Unlike other ERBB family members, HER2 does not require a direct ligand for activation; instead, it acts as a preferred co-receptor, forming heterodimers with other ERBB proteins (e.g., EGFR/ERBB1, HER3/ERBB3, and HER4/ERBB4) [86]. These heterodimers potentiate downstream signaling, amplifying critical pathways, such as the PI3K/AKT [87] and MAPK/ERK [88] cascades, which promote cell proliferation, survival, and invasion.

HER2’s ability to enhance signaling efficiency makes it essential in normal cellular function and development. However, its overexpression or gene amplification is implicated in various cancers, particularly HER2+ breast cancer, where it drives aggressive tumor growth and contributes to therapy resistance. HER2 overexpressed or amplified in approximately 25–30% of breast cancers [40], as well as in subsets of gastric [89], ovarian [90], and other solid tumors [91]. This overexpression correlates with aggressive disease phenotypes, increased metastasis, and reduced survival.

This dual role in normal physiology and disease underscores HER2’s significance as both a biological marker and a therapeutic target in oncology.

The mechanism of action for HER2 blockade focuses on interrupting the signaling pathways mediated by HER2 that are frequently overexpressed or amplified in aggressive cancers, such as breast and gastric cancers [92]. In normal cells, HER2-mediated signaling is tightly regulated; however, in cancers, HER2 overexpression results in its constitutive activation or ligand-independent dimerization, leading to dysregulated signaling.

This dysregulation drives the hyperactivation of downstream oncogenic pathways, such as the PI3K/AKT/mTOR pathway [88], which promotes cell survival and resistance to apoptosis, and the RAS/MAPK/ERK pathway [93], which supports excessive cell proliferation. These pathways work together to create a highly favorable environment for tumor growth, angiogenesis, and metastatic spread. Additionally, HER2 amplification is often associated with resistance to standard therapies, making it a critical therapeutic target.

By blocking HER2-mediated signaling, therapies aim to restore control over these pathways, suppressing tumor progression and enhancing the susceptibility of cancer cells to other treatments, ultimately improving patient outcomes.

Various approaches have been developed to achieve HER2 blockade, each targeting specific aspects of HER2 biology.

#### 3.1.1. Monoclonal Antibodies

Trastuzumab and pertuzumab are two monoclonal antibodies approved by the U.S. Food and Drug Administration (FDA) that target the extracellular domain of HER2.

Trastuzumab (Herceptin) binds to subdomain IV of the extracellular domain of the HER2 receptor. By blocking this region, trastuzumab inhibits HER2 from forming heterodimers with other members of the ERBB receptor family, thereby disrupting downstream signaling pathways that drive cell proliferation and survival [94]. In addition, trastuzumab triggers antibody-dependent cellular cytotoxicity (ADCC) [95], marking HER2-overexpressing tumor cells for immune-mediated destruction by recruiting natural killer (NK) cells and other immune effectors.

Clinically, trastuzumab is approved for the treatment of HER2-positive breast cancer, including early-stage disease, where it is often used in adjuvant or neoadjuvant settings to reduce recurrence risk, and metastatic BC, typically in combination with chemotherapy [96] or other HER2-targeted therapies [97]. It is also approved for HER2-positive metastatic gastric or gastroesophageal junction cancer [98], offering a targeted treatment option for these malignancies. Since its introduction, trastuzumab has revolutionized the management of HER2-positive cancers, significantly improving outcomes such as overall survival and disease-free survival, establishing itself as a cornerstone of HER2-targeted therapy.

Pertuzumab (Perjeta) binds specifically to subdomain II of the HER2 extracellular domain, effectively blocking the formation of HER2/HER3 heterodimers, which are critical drivers of HER2-mediated signaling and tumorigenesis [79]. By targeting this unique mechanism, pertuzumab complements the action of trastuzumab, which binds to a different region of HER2 (subdomain IV). When used in combination with trastuzumab and chemotherapy, pertuzumab leverages their synergistic effects, enhancing antitumor efficacy [99]. This combination has become a standard-of-care in the treatment of HER2-positive metastatic BC, significantly improving progression-free and overall survival compared to trastuzumab and chemotherapy alone.

#### 3.1.2. Small Molecule Tyrosine Kinase Inhibitors (TKIs)

Small molecules, such as lapatinib and tucatinib, target the intracellular kinase domain of HER2, acting as competitive inhibitors of ATP binding to block downstream signaling cascades [80]. These agents are particularly effective in disrupting HER2-driven tumor growth. Dual HER2/EGFR inhibitors, like lapatinib, offer added versatility, especially in tumors expressing multiple HER family members. Notably, the FDA has approved tucatinib for use in combination with immunotherapy and/or chemotherapeutic agents in the treatment of advanced or metastatic HER2-positive breast cancer [100]. Additionally, lapatinib has demonstrated activity in HER2-driven tumors that are resistant to trastuzumab, providing an alternative therapeutic option in such cases [101].

#### 3.1.3. Antibody-Drug Conjugates (ADCs)

ADCs represent a revolutionary class of targeted cancer therapeutics, combining the precision of monoclonal antibodies with the potent effects of cytotoxic agent [81]. Their mechanism of action involves a series of well-orchestrated steps, starting with the selective targeting of tumor cells. The monoclonal antibody component of ADCs is engineered to recognize and bind to specific antigens, such as HER2, CD20, or CD33, which are predominantly or exclusively expressed on the surface of cancer cells. This precise target minimizes off-target effects and reduces the risk of damage to normal tissues.

Once the ADC binds to its target antigen, the complex is internalized through receptor-mediated endocytosis [102], allowing the therapeutic payload to enter the tumor cell. Inside the cell, the ADC is trafficked to lysosomes, where the acidic environment and enzymatic activity degrade the antibody or linker [103]. This degradation is a crucial step in releasing the cytotoxic drug. The linker connecting the antibody and the drug is specifically designed to respond to intracellular conditions. Cleavable linkers, sensitive to low pH, proteases, or glutathione levels, release the payload in a targeted manner [104], [105]. Non-cleavable linkers, on the other hand, require complete antibody degradation for payload release, ensuring drug delivery exclusively within the tumor cell.

Once released, the cytotoxic payload exerts its effect by targeting critical cellular processes. Most ADC payloads are extremely potent, capable of killing tumor cells at picomolar concentrations. The common mechanisms of action include microtubule inhibition [106], as seen with agents like auristatins [107] and maytansinoids [108], which disrupt mitotic spindle formation and induce cell cycle arrest and apoptosis. Another mechanism involves DNA-damaging agents, such as calicheamicins [109] and pyrrolobenzodiazepines [110], which cause DNA breaks, ultimately leading to cell death.

In some cases, ADCs exhibit a beneficial “bystander effect”. The released payload can diffuse into neighboring cells, killing antigen-negative cells within the TME [111]. This is particularly advantageous in heterogeneous tumors where not all cells express the target antigen. Additionally, the antibody component of ADCs may retain the ability to mediate immune responses, such as antibody-dependent cellular cytotoxicity (ADCC) or complement-dependent cytotoxicity (CDC), further enhancing their therapeutic potential.

ADCs offer several advantages over conventional therapies [112]. Their high specificity significantly reduces systemic toxicity, and their potent payloads can effectively target resistant or dormant tumor cells. Moreover, ADCs are amenable to combination therapies with other modalities, including immunotherapies, providing a versatile platform for cancer treatment.

Notable examples of approved ADCs include trastuzumab emtansine (T-DM1), which targets HER2 and delivers the microtubule inhibitor DM1 [113], brentuximab vedotin, which targets CD30 with the MMAE payload for Hodgkin lymphoma [114], and sacituzumab govitecan, which targets Trop-2 with SN-38, a topoisomerase I inhibitor [115]. This modular design allows ADCs to be tailored to various cancers, enhancing their versatility and therapeutic potential.

#### 3.1.4. Challenges and Future Directions

Despite the success of HER2-targeted therapies, resistance mechanisms, such as receptor mutations [116], alternative signaling pathway activation [117], and phenotypic switching [118], remain significant challenges. Future research is focused on understanding resistance biology, optimizing combination therapies, and integrating emerging technologies, like artificial intelligence, to identify predictive biomarkers. Novel approaches, including HER2-specific CAR-T cells and nanotechnology-based delivery systems, are also in development to improve specificity and minimize systemic toxicities.

The continuous evolution of HER2 block strategies holds promise for more effective, personalized treatment options and better outcomes for patients with HER2-positive malignancies.

### 3.2. Immune Checkpoint Inhibition: Revolutionizing Cancer Immunotherapy

The advent of ICIs has revolutionized the field of oncology, introducing a groundbreaking approach that leverages the body’s immune system to combat cancer [119,120]. These therapies specifically target key regulatory pathways in T-cells that are often hijacked by tumors to evade immune detection and destruction. By blocking these inhibitory signals, ICIs restore and enhance immune system activity, enabling a more effective antitumor response.

This innovative therapeutic strategy represents a paradigm shift in cancer treatment, particularly for malignancies that were previously resistant to conventional modalities, such as chemotherapy and radiation. ICIs have not only demonstrated efficacy in extending survival rates but have also redefined the standard-of-care in a variety of cancers, including melanoma [121], non-small cell lung cancer [122], renal cell carcinoma [123], and breast cancer [124]. As our understanding of tumor–immune interactions deepens, ICIs continue to hold promises for expanding their application across a broader spectrum of cancers, including those with complex immunosuppressive microenvironments.

#### 3.2.1. Mechanisms of Immune Checkpoint Inhibition

Immune checkpoints are intrinsic regulators of the immune response that maintain self-tolerance and prevent autoimmunity. However, tumors upregulate these pathways to suppress T-cell activation and escape immune destruction. The two most extensively studied immune checkpoint molecules are programmed death 1 (PD-1) [125] and cytotoxic T-lymphocyte-associated protein 4 (CTLA-4) [126].

#### 3.2.2. PD-1/PD-L1 Axis

PD-1, a co-inhibitory receptor expressed primarily on activated T-cells, plays a critical role in maintaining immune homeostasis by modulating T-cell activity. Its ligands, PD-L1 and PD-L2, are frequently overexpressed on tumor cells and tumor-infiltrating immune cells as a mechanism to evade immune surveillance. The interaction between PD-1 and its ligands inhibits T-cell receptor (TCR) signaling, impairing the ability of T-cells to proliferate, secrete cytokines, and mount effective antitumor responses [127]. This immune suppression contributes to a state of T-cell exhaustion, a hallmark of the TME in many cancers [128].

Monoclonal antibodies targeting this axis have emerged as a cornerstone of immune checkpoint blockade therapies. Pembrolizumab and nivolumab, which specifically bind to PD-1, prevent its interaction with both PD-L1 and PD-L2, thereby restoring T-cell functionality [129,130]. Similarly, agents such as atezolizumab and durvalumab target PD-L1, disrupting its binding to PD-1 and additionally blocking its interaction with CD80, another immune regulatory receptor [131]. By reactivating exhausted effector T-cells, these therapies reinvigorate the immune response against tumors, leading to durable anticancer effects in several malignancies. The clinical success of PD-1/PD-L1 inhibitors highlights the significance of this pathway as a therapeutic target, spurring ongoing efforts to enhance efficacy through combination strategies and overcome resistance mechanisms [132].

#### 3.2.3. CTLA-4 Pathway

CTLA-4 competes with the co-stimulatory molecule CD28 for binding to B7 ligands (CD80/CD86) expressed on antigen-presenting cells (APCs) [133]. By outcompeting CD28, CTLA-4 attenuates T-cell activation during the priming phase in lymphoid organs. This mechanism is crucial for maintaining immune homeostasis and preventing excessive immune responses.

Ipilimumab, a monoclonal antibody targeting CTLA-4, disrupts this inhibitory pathway by blocking the interaction between CTLA-4 and B7 ligands [83]. This blockade restores CD28-mediated co-stimulation, thereby enhancing T-cell activation and proliferation. By reinvigorating T-cell responses, ipilimumab promotes a robust antitumor immune response, making it a pioneering therapeutic agent in the field of immune checkpoint inhibition for cancer immunotherapy.

#### 3.2.4. Clinical Applications

ICIs have shown remarkable efficacy in treating a variety of cancers, including breast cancer [134]. Their success has led to rapid regulatory approvals and integration into standard-of-care regimens.

Breast cancer, historically regarded as less immunogenic compared to malignancies like melanoma or lung cancer [135], has emerged as a critical focus for immune checkpoint inhibition strategies. Among its subtypes, TNBC stands out due to its higher levels of immune cell infiltration [136] and the elevated expression of immune checkpoint molecules, such as PD-L1 [137], making it particularly responsive to these therapies. Clinical trials have shown that ICIs, especially those targeting the PD-1/PD-L1 pathway, can significantly enhance antitumor immunity in breast cancer, particularly when used in combination with chemotherapy [138]. This synergistic approach leverages chemotherapy to enhance antigen presentation and alleviate immune suppression within the TME. However, the effectiveness of ICIs in HR+ and HER2-positive breast cancers remains suboptimal, with mixed outcomes [139].

The limited response of patients with HR+ breast cancer to ICIs is primarily due to low TIL levels, low TMB, and low PD-L1 expression [140]. In the context of HER2-positive breast cancer, the strong antitumor immune response driven by anti-HER2 antibodies—mediated through antibody-dependent cellular cytotoxicity (ADCC) and antibody-dependent cellular phagocytosis (ADCP)—may reduce the potential benefit of adding anti-PD-L1 antibodies to standard therapy [141].

This underscores the urgent need for reliable biomarkers to predict treatment responses and innovative approaches to overcome therapeutic resistance.

The common ICI resistance mechanisms are tumor-intrinsic resistance, tumor-extrinsic factors further, and the gut microbiome.

Tumor-intrinsic resistance mechanisms include disruptions in antigen presentation, allowing cancer cells to evade immune detection. Mutations or the downregulation of β2-microglobulin (B2M) and MHC-I molecules reduce tumor visibility to T-cells, while defects in antigen-processing machinery further impair the presentation of neoantigens [142]. Some tumors evolve to eliminate highly immunogenic neoantigens, making them less recognizable by the immune system [143]. Additionally, tumors can upregulate alternative immune checkpoints, such as TIM-3, LAG-3, TIGIT, and VISTA, effectively bypassing the PD-1/CTLA-4 blockade. Oncogenic signaling pathways also play a role; for example, the activation of the WNT/β-catenin pathway reduces dendritic cell infiltration [144], PTEN mutations trigger the PI3K-AKT pathway to suppress immune activity [145], and JAK1/JAK2 mutations disrupt IFN-γ signaling, diminishing T-cell activation [146].

Tumor-extrinsic factors further contribute to resistance. The tumor microenvironment (TME) can become highly immunosuppressive due to the infiltration of regulatory T-cells (Tregs), myeloid-derived suppressor cells (MDSCs), and tumor-associated macrophages (TAMs), all of which suppress effector T-cell function. Elevated levels of TGF-β, IL-10, and VEGF further reinforce this immunosuppressive state [147]. Additionally, metabolic competition within the TME plays a role [148], as tumor cells outcompete immune cells for key nutrients, such as glucose and tryptophan, leading to T-cell exhaustion. Hypoxic conditions within tumors induce HIF-1α, promoting the expansion of Tregs and impairing cytotoxic T-cell function [148].

The gut microbiome also influences ICI efficacy [149], with certain bacterial species enhancing response, while others contribute to resistance. For instance, *Bacteroides fragilis* [150] and *Akkermansia muciniphila* [151] have been associated with improved ICI responses; whereas, *Blautia obeum* and *Roseburia intestinalis* have a negative impact on ICI treatment [152]. Epigenetic modifications add another layer of complexity, as DNA methylation and histone modifications can silence key genes involved in antigen presentation and immune responses. Additionally, tumors may develop adaptive resistance by dynamically altering immune checkpoint expression in response to therapy.

Current research efforts aim to expand the utility of ICIs in breast cancer by integrating them with targeted therapies, therapeutic vaccines, and other immune-modulating agents [153]. These combinations are designed to optimize therapeutic outcomes and extend the benefits of ICIs across all breast cancer subtypes.

Combination therapies have emerged as a transformative strategy to overcome resistance to ICIs [154]. By targeting distinct mechanisms of immune evasion, these approaches enhance antitumor immunity and expand the therapeutic potential of ICIs across a broad spectrum of cancer types.

Checkpoint combination therapy leverages the dual blockade of immune checkpoints, such as PD-1/PD-L1 and CTLA-4 [155], which regulate distinct stages of T-cell activation. CTLA-4 inhibition promotes the priming of naïve T-cells, while the PD-1/PD-L1 blockade restores the function of effector T-cells suppressed within the TME. This complementary mechanism has proven to be particularly effective in enhancing immune-mediated tumor control.

Nivolumab, a monoclonal antibody targeting PD-1 [156], and ipilimumab, a monoclonal antibody targeting CTLA-4 [157], are among the most extensively studied ICIs. These agents have demonstrated significant antitumor activity across various malignancies, including melanoma, renal cell carcinoma, non-small cell lung cancer, and hepatocellular carcinoma [158].

The combination of nivolumab and ipilimumab has shown synergistic effects in numerous cancers, offering improved response rates compared to monotherapy. Emerging evidence also supports the potential efficacy of this combination in breast cancer [159,160], a historically less immunogenic malignancy. Recent studies have highlighted significant improvements in response rates and outcomes when nivolumab and ipilimumab are used together in specific BC subtypes, such as TNBC. This underscores the versatility and expanding applicability of checkpoint combination therapies in overcoming resistance and improving outcomes in diverse oncological settings.

Combining ICIs with chemotherapy has also shown promise [161]. Chemotherapy induces immunogenic cell death, releasing tumor antigens that prime the immune system and modulating the TME by depleting immunosuppressive cells, like myeloid-derived suppressor cells (MDSCs). In TNBC, the combination of atezolizumab (anti-PD-L1) with nab-paclitaxel (chemotherapy) prolonged progression-free survival among patients with metastatic TNBC, establishing it as a key treatment for PD-L1-positive TNBC [162].

Radiotherapy combined with ICIs is another effective strategy. Radiotherapy enhances tumor antigen presentation and T-cell infiltration by inducing DNA damage and pro-inflammatory signaling, transforming immunologically “cold” tumors into “hot” ones [163]. The concept of “hot” and “cold” tumors provides a framework for understanding the variability in clinical responses to cancer treatment. Hot tumors are characterized by a robust immune response and typically respond well to therapies, particularly immunotherapies. In contrast, cold tumors exhibit minimal immune infiltration and poor responsiveness to treatment, posing a significant challenge in oncology. This distinction underscores the need for tailored therapeutic strategies to convert cold tumors into hot ones, thereby enhancing their susceptibility to immunotherapeutic approaches [164]. For instance, in non-small cell lung cancer (NSCL), pembrolizumab (anti-PD-1) combined with radiotherapy exploits the “abscopal effect”, where localized radiation triggers systemic immune activation, leading to improved outcomes [165]. The combination of pembrolizumab and radiotherapy (RT) has been shown to be safe and exhibits promising efficacy in patients with metastatic TNBC [166], even in those with poor prognoses and without selection based on PD-L1 expression. This approach highlights the potential of combining ICIs with RT to enhance therapeutic outcomes in hard-to-treat cancers, like TNBC.

Cancer vaccines and ICIs provide another promising avenue [167]. Vaccines introduce tumor-specific antigens to the immune system, priming T-cells to recognize and attack cancer cells. Combining vaccines with ICIs amplifies immune responses by preventing T-cell exhaustion. In metastatic melanoma, a personalized neoantigen vaccine combined with pembrolizumab achieved durable responses [168], and ongoing trials are exploring similar combinations in colorectal [169] and breast cancers [170].

The synergistic effect of vaccines with ICIs have been proved in several types of cancer including in BC [171] pancreatic ductal adenocarcinoma [172], large-sized glioma tumors [173], HPV associated tumors [174], melanoma, and prostate cancer [175].

ICIs with oncolytic viruses (OVs) represent [176] a novel strategy. OVs selectively infect tumor cells, causing lysis and releasing tumor antigens that stimulate immune responses. When paired with ICIs, they enhance T-cell activation and mitigate immune escape. Carter et al. (2022) demonstrated that OVs significantly reduced cell viability in organoid cultures derived from breast cancer tissue [177]. Similarly, Arab et al. (2019) reviewed the potential of OVs and phage display as preventive vaccines in breast cancer [178]. However, no study to date has combined these two therapeutic strategies for breast cancer treatment. Notably, Talimogene laherparepvec (T-VEC), an oncolytic herpesvirus, demonstrated enhanced response rates when combined with ipilimumab in melanoma patients compared to ipilimumab alone [179].

Epigenetic modifiers and ICIs are also gaining attention. Drugs like histone deacetylase (HDAC) inhibitors and DNA methyltransferase inhibitors increase tumor immunogenicity by upregulating tumor antigen expression and enhancing MHC presentation. Preclinical studies have shown that the HDAC inhibitor entinostat enhances the antitumor effect of PD-1 inhibition in breast [180], lung and renal [181], and bladder cancer [182].

ICIs and cytokine therapies use cytokines such as IL-2 and IL-15 to boost T-cell proliferation and activity, while ICIs sustain their effector function. For example, bempegaldesleukin, an IL-2 variant, combined with nivolumab, is being evaluated in advanced solid tumors, showing potential to enhance response rates in early-phase trials [183].

Finally, modulating the gut microbiome represents a promising frontier in enhancing the efficacy of ICIs [184]. A favorable gut microbiome has been shown to boost both systemic and antitumor immunity, contributing to better outcomes in responding patients [185]. Specific bacterial species, such as Akkermansia muciniphila, have been linked to improved responses in ICI-treated patients [186]. Moreover, a growing body of research is investigating strategies to modulate the gut microbiota to overcome resistance to immune checkpoint blockades in cancer immunotherapy [82].

### 3.3. Chimeric Antigen Receptor Macrophages (CAR-M) as a Novel Immunotherapeutic Approach in Breast Cancer

Breast cancer remains a leading cause of cancer-related mortality worldwide, necessitating the development of innovative therapeutic strategies to improve patient outcomes. Chimeric antigen receptor macrophages (CAR-M) have emerged as a novel immunotherapeutic approach, leveraging the innate phagocytic and antigen-presenting capabilities of macrophages to target and eliminate tumor cells. Unlike chimeric antigen receptor T-cells (CAR-T), which primarily exert their effects through cytotoxic lymphocyte-mediated tumor killing, CAR-Ms harness the inherent plasticity and tumor-infiltrating ability of macrophages to overcome the immunosuppressive TME [84].

Recent studies have demonstrated that CAR-M therapy can effectively reprogram the TME by enhancing antigen presentation, promoting pro-inflammatory responses, and disrupting the immune evasion mechanisms of breast cancer cells [187]. Engineered macrophages expressing CARs specific to tumor-associated antigens, such as HER2 or epithelial cell adhesion molecule (EpCAM), exhibit robust phagocytic activity and direct tumoricidal effects [188]. Additionally, CAR-Ms have been shown to stimulate adaptive immune responses by recruiting and activating T-cells, thereby potentiating long-term antitumor immunity.

A key advantage of CAR-M therapy in breast cancer is its ability to overcome resistance to traditional immunotherapies. Solid tumors, including breast cancer, often develop mechanisms to evade immune recognition through the upregulation of immune checkpoint molecules, secretion of immunosuppressive cytokines, and recruitment of regulatory immune cells. CAR-Ms counteract these strategies by modulating the TME, enhancing antigen presentation, and stimulating innate immune responses. Preclinical studies have indicated that CAR-M therapy can effectively reduce the tumor burden in murine models of breast cancer by targeting endothelial growth factor receptor-2 (VEGFR2) [187], HER-2 [188], or other receptors, paving the way for clinical translation.

Challenges remain in optimizing CAR-M therapy for clinical application, including ensuring macrophage persistence, complex TME, the delivery of CAR M in tumor sites, and achieving efficient in vivo expansion [189]. Strategies such as the use of induced pluripotent stem cell-derived macrophages (iPSC-Ms) [190], enhanced gene editing techniques [191], and combination approaches with immune checkpoint inhibitors [192] are being explored to enhance CAR-M efficacy. As research advances, CAR-M therapy holds great promise as a transformative approach in breast cancer treatment, offering a novel avenue for targeting resistant and metastatic disease.

Future clinical trials will be essential to evaluate the safety, efficacy, and long-term benefits of CAR-M therapy in breast cancer patients. The integration of CAR-M with existing treatment modalities, including chemotherapy, radiation, and immune checkpoint blockade, may further enhance therapeutic outcomes and provide new hope for patients with advanced breast cancer.

### 3.4. Advantages and Disadvantages of Immunotherapy for Breast Cancer

Immunotherapy for breast cancer has shown promise, particularly in triple-negative breast cancer (TNBC), but its effectiveness varies depending on the subtype. Below is a breakdown of its advantages and disadvantages.

#### 3.4.1. Advantages of Immunotherapy for Breast Cancer

- Enhanced tumor-specific response: unlike chemotherapy, which affects both cancerous and healthy cells, immunotherapy activates the immune system to specifically target cancer cells [193].
- Long-lasting immune memory: some immunotherapies, particularly immune checkpoint inhibitors (ICIs), can induce long-term immune memory, reducing the chances of recurrence.
- Reduced systemic toxicity: compared to chemotherapy, immunotherapies often have fewer severe side effects, as they do not directly damage healthy tissues [194].
- Effectiveness in triple-negative breast cancer (TNBC): TNBC lacks hormone receptors and HER2, making it resistant to targeted therapies. However, immune checkpoint inhibitors (e.g., anti-PD-1/PD-L1) combined with chemotherapy have shown efficacy in TNBC [195].
- Potential for combination therapy: immunotherapy can be combined with chemotherapy [195], targeted therapy, or radiotherapy to improve treatment response [196].
- Personalized treatment approach: advances in biomarkers (e.g., PD-L1 expression, tumor mutational burden) enable patient selection for immunotherapy, increasing its effectiveness in suitable candidates [197].

#### 3.4.2. Disadvantages of Immunotherapy for Breast Cancer

- Limited efficacy in some subtypes: immunotherapy is most effective in TNBC, but hormone receptor-positive (HR+) and HER2-positive breast cancers tend to have lower immunogenicity, making them less responsive [140].
- Immune-related adverse events (irAEs): the overactivation of the immune system can lead to autoimmune-like side effects, such as colitis, pneumonitis, hepatitis, and thyroid dysfunction [198].
- High cost of treatment: immunotherapies, particularly monoclonal antibodies and immune checkpoint inhibitors, are expensive and may not be accessible to all patients [199].
- Development of resistance: some tumors develop mechanisms to evade immune detection, leading to primary or acquired resistance [200].
- Delayed response or pseudo-progression: some patients experience an initial increase in tumor size (pseudo-progression) before seeing a clinical response, making treatment evaluation challenging [201].
- Need for biomarker identification: the effectiveness of immunotherapy depends on biomarkers like PD-L1 expression and tumor mutational burden, which may not always be present in breast cancer patients [202].

In conclusion, immunotherapy represents a significant advancement in breast cancer treatment, especially for TNBC, but challenges like immune-related toxicity, cost, and limited efficacy in certain subtypes remain. Future research aims to refine biomarker-driven approaches and combination strategies to improve outcomes.

## 4. Anticancer Vaccines in Breast Cancer

Anticancer vaccines are emerging as a highly promising and innovative approach to stimulating the immune system to mount strong, long-lasting antitumor responses [203]. By harnessing the body’s natural immune mechanisms, these vaccines aim to educate the immune system to recognize and target cancer cells more effectively. With the potential to provide both preventive and therapeutic benefits, anticancer vaccines offer hope for achieving more durable remissions, improving patient outcomes, and ultimately enhancing survival rates. Researchers are continuously exploring various strategies, including personalized cancer vaccines, that can further refine immune responses for even greater precision and efficacy in combating different types of cancer. Different types of vaccines have been utilized for the treatment of breast cancer (Table 4).

### 4.1. Protein- or Whole-Cell-Based Vaccines

Protein-based and whole-cell-based vaccines represent powerful approaches in cancer immunotherapy, designed to stimulate both the humoral (antibody-mediated) and cellular (T-cell-mediated) arms of the immune system [209]. These vaccines aim to prime the immune system to recognize and attack cancer cells by targeting tumor-associated antigens (TAAs) that are expressed on the surface of the tumor [210]. Both types of vaccines offer distinct advantages in terms of their antigenic coverage and ability to induce a robust and sustained immune response.

These vaccines use full-length TAAs in their native form to stimulate the immune system [211]. By introducing complete proteins, which may be naturally expressed on the surface of tumor cells [210], these vaccines are designed to elicit both humoral responses (antibodies) and cellular responses (cytotoxic T lymphocytes, or CTLs) against cancer cells. The inclusion of full-length TAAs ensures that the immune system can recognize a more comprehensive representation of the target antigen, which may improve the effectiveness of the immune response.

HER2 protein-based vaccines have shown promise in preclinical models and early-phase clinical trials [212]. These vaccines have demonstrated safety profiles and the ability to provoke an immune response, including the generation of HER2-specific antibodies and T-cell activation [213]. As a result, they offer potential as a therapeutic vaccine for HER2-positive cancers, especially in combination with other treatment modalities, like monoclonal antibodies [214] or chemotherapy [215]. HER2 vaccine have been combined with PD-L1 vaccine obtaining synergistic tumoricidal effect [216].

Whole-cell vaccines utilize either autologous (patient-derived) or allogeneic (donor-derived) tumor cells [217] to provide a wide variety of tumor antigens that can stimulate a broad immune response [204]. Because tumors are often heterogeneous, meaning that they may express a diverse array of antigens [218], whole-cell vaccines can present a more comprehensive set of targets than single-antigen vaccines [219]. This diversity may help overcome the challenge of tumor variability and resistance, potentially improving the efficacy of the vaccine across different tumor subtypes.

Vaccines with autologous tumor cells use cells derived from the patient’s own tumor, which are typically harvested, treated to enhance their immunogenicity (e.g., through irradiation or genetic modification), and then, reintroduced into the patient [220]. This approach allows for the presentation of tumor antigens that are specific to the individual’s cancer, thereby increasing the likelihood of a targeted immune response.

Vaccines with allogeneic tumor cells vaccines use tumor cells from a different patient, which can provide a broader spectrum of antigens for immune recognition [204]. These vaccines may offer advantages in situations where autologous tumor cell preparation is not feasible or when aiming to create a standardized vaccine for multiple patients with similar cancer types [204].

Whole-cell vaccines have the potential to activate multiple components of the immune system, including dendritic cells, macrophages, and various T-cell populations, thereby promoting both innate and adaptive immunity [221]. Moreover, their ability to expose the immune system to a wide array of tumor antigens may reduce the likelihood of immune escape and tumor relapses, making them a promising candidate for use in cancer immunotherapy.

However, whole-cell vaccines face several limitations, including challenges in obtaining material from hard-to-access tumors and the potential inclusion of self-antigens. Moreover, they have been associated with suboptimal antigen-presenting cell (APC) uptake, inefficient antigen cross-presentation, and the weak activation of CD8+ T-cell responses [222].

### 4.2. DNA and RNA Vaccines

Nucleic acid-based vaccines, including DNA and RNA vaccines, represent a revolutionary approach in cancer immunotherapy by utilizing the body’s own cells to produce TAAs and trigger targeted immune responses [223]. These vaccines rely on the introduction of genetic material—either DNA or RNA—into the body, enabling host cells to transcribe and translate these genetic instructions into antigenic proteins. The immune system then recognizes these proteins as foreign, stimulating both humoral and cellular immune responses that can target and eliminate cancer cells expressing these specific antigens.

#### 4.2.1. DNA Vaccines

DNA-based vaccines consist of plasmids—circular DNA molecules—that encode for specific TAAs. When administered, the plasmid DNA is taken up by cells in the body, which then express the encoded tumor antigens [224]. This process activates the immune system to produce a targeted response, including the generation of antibodies and cytotoxic T-cells that can identify and attack tumor cells expressing the antigen [225]. DNA vaccines are highly customizable, enabling the inclusion of multiple antigens or immune-modulating sequences, which enhances their ability to stimulate a broad and effective immune response.

While DNA vaccines have shown potential in preclinical models, their application in cancer therapy has faced challenges related to efficient delivery and immunogenicity [225]. Researchers are exploring various strategies to improve these vaccines, including using advanced delivery systems, like electroporation [226] or lipid nanoparticles [227], to enhance the uptake of DNA by host cells. Despite these challenges, DNA vaccines remain a promising area of research, particularly for cancers that express unique or novel antigens that may be effectively targeted using personalized approaches.

#### 4.2.2. RNA Vaccines

RNA-based vaccines, particularly messenger RNA (mRNA) vaccines, have recently gained significant attention for their ability to rapidly induce potent immune responses. Unlike DNA vaccines, which require transcription within the host cell, mRNA vaccines deliver the genetic blueprint for the antigen directly in the form of RNA, which then instructs the host cells to produce the corresponding antigenic protein [205]. Once expressed, these antigens are recognized by the immune system, leading to the activation of both B cells (to produce antibodies) and T-cells (to eliminate infected or cancerous cells) [228].

mRNA vaccines have made substantial progress, particularly with the success of COVID-19 vaccines [229], leading to increased interest in their potential applications in cancer immunotherapy [230]. Preclinical and clinical studies have demonstrated that mRNA vaccines can elicit strong immune responses against tumor antigens [231], such as HER2, a well-known oncogene overexpressed in various cancers [232], including breast cancer [233]. These studies have shown that mRNA vaccines can effectively activate immune cells like dendritic cells and cytotoxic T lymphocytes (CTLs) that target and destroy HER2-expressing cancer cells.

One of the key advantages of mRNA vaccines is their ability to be rapidly designed and manufactured [234], allowing for quick adaptation to new tumor antigens or mutations. Moreover, mRNA vaccines do not require live virus vectors [235], making them less likely to provoke unwanted immune responses. The flexibility and speed with which mRNA vaccines can be produced have made them an attractive platform for personalized cancer vaccines, particularly in treating cancers with specific genetic mutations or alterations.

The development of both DNA and RNA vaccines is an area of intense research, with ongoing clinical trials exploring their use in various cancer types, including breast [233,236], melanoma [237], and lung [238] cancers. These nucleic acid-based vaccines offer the promise of targeted, highly effective treatments with the potential for long-term immunity against tumor recurrence. As technology continues to evolve, advancements in delivery systems, adjuvants, and antigen selection will likely enhance the potency and efficacy of these innovative vaccine platforms, providing new hope for cancer patients.

### 4.3. Dendritic Cell (DC) Vaccines

Dendritic cell (DC) vaccines represent a sophisticated and promising strategy in cancer immunotherapy, leveraging the powerful antigen-presenting capabilities of dendritic cells to stimulate robust, targeted immune responses against cancer cells [206]. Dendritic cells play a central role in the immune system by capturing, processing, and presenting antigens to T-cells, which then initiate specific immune responses to target and eliminate tumor cells. By harnessing this natural process, DC vaccines aim to enhance the body’s ability to recognize and destroy cancer cells.

The process of creating DC vaccines begins with the isolation of dendritic cells from the patient’s own blood (autologous DCs). These cells are then exposed to tumor antigens—either derived from the patient’s own tumor cells or from specific TAAs that are known to be present in the patient’s cancer. The dendritic cells internalize these antigens, process them, and present them on their surface, effectively “training” them to recognize cancer cells that express these same antigens. Once loaded with the tumor-specific information, these antigen-presenting cells are reintroduced into the patient’s body, where they can activate both the innate and adaptive arms of the immune system. Specifically, the dendritic cells stimulate T-helper cells and cytotoxic T lymphocytes (CTLs) to recognize and target the cancer cells that display the same antigens.

The primary goal of DC vaccines is to boost the immune system’s ability to specifically identify and destroy cancer cells, while minimizing damage to normal, healthy tissue. By reprogramming the dendritic cells to focus on the tumor antigens, these vaccines aim to create a tailored immune response that is highly specific to the individual’s cancer.

One of the most well-known success stories in DC vaccine development is Sipuleucel-T, a vaccine approved by the U.S. Food and Drug Administration (FDA) for the treatment of prostate cancer [239]. Sipuleucel-T marked a significant milestone in the field of cancer immunotherapy as the first FDA-approved dendritic cell-based vaccine. This vaccine is created by collecting a patient’s dendritic cells, exposing them to a fusion protein that includes a prostatic acid phosphatase (PAP) antigen and then reintroducing the activated cells back into the patient [240]. Sipuleucel-T has demonstrated efficacy in prolonging survival in patients with advanced prostate cancer and has helped validate the potential of dendritic cell-based vaccines in cancer treatment [241].

Sipuleucel-T’s success has opened the door for similar approaches in other cancer types, including breast cancer. Researchers are now investigating the potential of DC vaccines in treating breast cancer [242], particularly in patients with specific tumor antigens, such as HER2 [243], mucin 1 (MUC1) [244], or other tumor-associated markers that could be targeted through dendritic cell-based strategies. While still in the early stages of clinical development for BC, these studies are promising, with the hope of creating personalized vaccines that could significantly improve patient outcomes by enhancing the immune system’s ability to fight the tumor.

The potential of dendritic cell vaccines extends beyond prostate cancer to a wide range of malignancies, including breast, melanoma, and lung cancers. Several clinical trials are exploring how to optimize the process of generating these vaccines, including strategies to increase the potency of the dendritic cells, improve antigen loading, and enhance the overall immune response. Additionally, researchers are investigating ways to combine DC vaccines with other forms of cancer therapy, such as ICIs [245], chemotherapy [246], or targeted therapies [247], to create more comprehensive treatment regimens that further enhance the antitumor immune response.

Challenges remain in terms of the cost, complexity, and scalability of manufacturing these personalized vaccines [248]. However, advances in biotechnology, including improvements in dendritic cell generation, antigen identification, and vaccine delivery methods, are steadily overcoming these hurdles, bringing us closer to more widespread use of DC vaccines in clinical settings.

In conclusion, dendritic cell vaccines represent a promising and evolving approach to cancer immunotherapy. By harnessing the natural power of dendritic cells to stimulate specific and potent immune responses, these vaccines offer the potential for targeted, long-lasting cancer treatment. The success of Sipuleucel-T in prostate cancer has provided a roadmap for the development of similar therapies in other cancers, including breast cancer, and ongoing research continues to refine these strategies to improve their effectiveness and accessibility for patients worldwide.

### 4.4. Viral Vector-Based Vaccines

Viral vector-based vaccines represent an innovative and effective approach in cancer immunotherapy by utilizing modified viruses to deliver TAA genes into host cells [207]. These viral vectors, often derived from adenoviruses or poxviruses [249], are engineered to carry genetic material that encodes specific antigens expressed by cancer cells. Once the viral vectors are introduced into the body, the host cells take up the genetic material, leading to the production of tumor antigens. The immune system then recognizes these antigens as foreign, triggering both humoral (antibody-mediated) and cellular (T-cell-mediated) immune responses to target and eliminate the cancer cells expressing the same antigens.

The use of viral vectors in vaccine development offers several advantages, including the ability to efficiently deliver genetic material to a wide range of cells, the potential to stimulate a strong and sustained immune response, and the ability to target multiple tumor antigens simultaneously. By mimicking a natural viral infection, these vaccines can effectively activate the immune system to recognize and fight tumor cells, even those that might otherwise evade detection.

One prominent example of a viral vector-based vaccine is PANVAC, a recombinant poxvirus vaccine designed to express two tumor-associated antigens: carcinoembryonic antigen (CEA) and mucin 1 (MUC1) [250]. CEA is a protein frequently overexpressed in a variety of cancers, including breast [251], colon [252], and pancreatic cancers [253], while MUC1 is a glycoprotein often aberrantly expressed in breast cancer [254]. The PANVAC vaccine utilizes a modified poxvirus vector to deliver the genetic instructions for these antigens to the immune system, with the aim of provoking an immune response that targets and eliminates cancer cells expressing CEA and MUC1.

PANVAC in combination with Docetaxel has undergone evaluation in clinical trials for breast cancer, with encouraging results demonstrating its ability to generate a robust immune response [255]. In these trials, the vaccine has shown potential to stimulate T-cell and antibody responses that target and attack cancer cells, contributing to tumor regression and improved patient outcomes. The continued development of PANVAC and other viral vector-based vaccines could provide a valuable therapeutic option, particularly for cancers that express well-characterized tumor antigens, like CEA and MUC1.

Viral vector-based vaccines are being actively explored for use in a wide range of cancers, and ongoing clinical trials are evaluating their safety, efficacy, and potential in combination with other cancer therapies. The flexibility of viral vectors allows for the inclusion of multiple tumor antigens or even immune-modulating genes, which can further enhance the specificity and potency of the immune response. Moreover, viral vectors can be engineered to overcome some of the challenges faced by other vaccine platforms, such as the need for efficient delivery systems or the risk of immune tolerance to certain antigens.

Researchers are also investigating the potential of combining viral vector vaccines with other immunotherapies, such as ICIs, to enhance the overall immune response and address immune suppression mechanisms within the TME. This combination approach could lead to more effective, long-lasting therapies for patients with difficult-to-treat cancers.

One significant limitation of viral vector-based vaccines is the development of anti-vector immunity after the initial immunization. This immune response can reduce the effectiveness of subsequent doses, leading to diminished efficacy when the same vector is re-administered for boosting [256].

### 4.5. Epitope-Based Vaccine Against Breast Cancer

Peptide-based vaccines are an innovative approach in cancer immunotherapy, using short chains of amino acids derived from TAAs [257] or tumor-specific antigens (TSAs) [208]. They can be developed to induce the immune system, specifically T-cell responses, to target and destroy cancer cells expressing these distinct antigens, while minimizing damage to healthy, normal tissues.

Various epitopes from different antigens associated with breast cancer have been utilized in the development of breast cancer vaccines, which have been tested in both preclinical and clinical studies (Table 5).

#### 4.5.1. HER2

Human epidermal growth factor receptor 2 (HER2), also known as ErbB2 or EGFR2, is a 185 kDa transmembrane glycoprotein and one of the members of the epidermal growth factor receptor family. HER2 plays a pivotal role in controlling cell growth and division. When this pathway is abnormally activated, it can lead to excessive cell proliferation, angiogenesis, and ultimately, tumor formation [271]. The overexpression or gene amplification of HER2 has been identified in various solid tumors, notably in 25–30% of primary breast cancers [40], where it is associated with poor prognosis and aggressive tumor behavior [41]. HER2 is not just a marker of tumorigenesis but is recognized as an oncogenic driver; therefore, HER2 has become a critical target for cancer therapy. However, low HER2 expression is a predictor of poor prognosis in TNBC [272].

The HER2 protein consists of four key domains: the extracellular domain (ECD), transmembrane domain (TMD), intracellular domain (ICD), and the C-terminal tail. The ECD, which is made up of approximately 620 amino acids, is responsible for ligand binding and is further divided into four subdomains. Subdomain I is involved in ligand binding, subdomain II in receptor dimerization, subdomain III serves as another ligand-binding region, and subdomain IV stabilizes the dimer and contains cysteine-rich regions [273].

The TMD is a single hydrophobic alpha-helix that spans the plasma membrane and is essential for receptor activation and stabilization. The ICD has kinase activity, crucial for signal transduction. When tyrosine residues are phosphorylated, the kinase domain triggers downstream signaling pathways, such as MAPK and PI3K/AKT, which drive cell proliferation and survival. The C-terminal tail, containing multiple tyrosine residues, serves as docking sites for signaling molecules upon phosphorylation, initiating critical intracellular signaling cascades [274].

**Figure 1:**
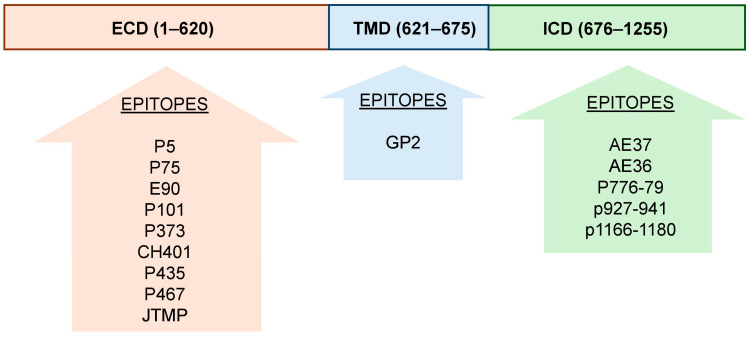
Epitopes utilized in epitope-based vaccines, mapped across the extracellular domain (ECD), transmembrane domain (TMD), and intracellular domain (ICD) of the HER-2 protein.

Most BC immunotherapies target the HER2 domains due to their pivotal role in cancer progression (Figure 1).

- Epitopes from HER2 extracellular domain

Several epitopes of the HER2 extracellular domain have been targeted in BC vaccine formulations.

The P5 and P435 epitopes are restricted to MHC class I molecules in BALB/c mice. Both epitopes, either individually or in combination, have been shown to elicit a robust cytotoxic T lymphocyte (CTL) response and inhibit tumor progression. These effects were observed when the epitopes were delivered using liposomes in various formulations, either as a vaccine or as an adjuvant delivery system. The studies were conducted in the TUBO mouse model of BC, which overexpresses the rat HER2/neu protein [275].

The CH401 epitope, spanning residues 167 to 175 of HER2, contains regions recognized by both CD4(+) and CD8(+) T-cells, playing a key role in suppressing the growth of Her-2/neu-expressing tumors [259]. A vaccine formulation incorporating the CH401 epitope, displayed on plant-derived virus-like particles (VLPs), was used to immunize subcutaneous BALB/c mice. Fourteen days after the third immunization, the mice were challenged with HER2+ mouse tumor cells (DDHER2) via subcutaneous injection in the right flank. The results demonstrated that the VLP-based vaccine induced a strong immune response, characterized by high titers of HER2-specific antibodies and enhanced cytotoxic activity of antisera against DDHER2 tumor cells. Tumor growth was significantly delayed in the vaccinated mice, leading to prolonged survival compared to naïve controls [276].

A multi-peptide vaccine targeting HER2 has been shown to be effective in preventing tumor progression in mice with preinvasive breast disease [277].

The peptides p101 and p373, MHC class II epitopes derived from the ECD of murine ErbB-2, were used in a mixture to immunize female MMTV-PyMT transgenic mice. These mice were subsequently challenged with a mouse mammary carcinoma (MMC) cell line, which was previously established from a spontaneous tumor harvested from neu-tg mice. The peptide-based vaccine completely suppressed the development of spontaneous breast tumors, with its efficacy strongly associated with robust antigen-specific T-cell and antibody responses [263].

Vincent et al. (2023) evaluated the efficacy of the MHC class I epitopes E75 (nelipepimut-S or NPS) and E90 in a multiepitope dendritic cell (DC) vaccine combined with monoclonal antibody therapy and chemotherapy for treating patients with HER2-overexpressing metastatic breast cancer. The rationale for dual targeting with E75 and E90 is based on preclinical findings showing that E90-specific immune responses enhance the antitumor effects of E75. The treatment in this study involved trastuzumab and vinorelbine, and autologous peptide-pulsed DCs—specifically CD34+-derived DCs incubated with E75 or E90. The results demonstrated that DC vaccination induced a robust immune response, characterized by a significant expansion of E75-specific CD8+ T-cells and an increased frequency of CD107+ and IFN-γ–secreting T-cells upon E75 and E90 stimulation. However, these immune responses did not show a direct correlation with clinical outcomes. The combination therapy stabilized disease in 46% of patients, with 4% achieving a partial response. Notably, one patient, who exhibited a strong T-cell response to the E75 epitope, survived for over 14 years post-treatment. A key limitation of the study was the lack of a control group that received only vinorelbine and trastuzumab without the vaccine. Nevertheless, the findings highlight the potential of this combination therapy in metastatic breast cancer and support the inclusion of the E75 and E90 peptides in a multiepitope HER2 vaccine formulation [260].

HERVaxx (IMU-131) is a B-cell peptide vaccine composed of a fusion of three epitopes (P467) from the extracellular domain of HER2/neu conjugated to the nontoxic diphtheria protein CRM197 with the adjuvant Montanide [261].

In a HER2/neu-expressing lung metastasis (D2F2/E2 cells) syngeneic BC mouse model, HerVaxx significantly reduced tumor growth, performing comparably to passive immunization with pertuzumab. When combined with the pertuzumab mimotope JTMP, that is, a 42-mer peptide, as pertuzumab’s binding epitope on Her-2/neu (amino acids 260–301) [278], HerVaxx also prevented lung metastases. Furthermore, active immunization with HerVaxx, JTMP, or their combination induced strong HER2-specific antibody responses, along with increased infiltration of CD4+ and CD8+ T-cells in lung metastases. These findings underscore the potential of active immunization strategies in bolstering immune responses against HER2-positive tumors [262].

In addition of preclinical studies, the efficacy of HERVax has been evaluated in clinical trial for gastric cancer [215] and stomach cancer [279,280].

Many studies suggest that peptide-based vaccines targeting the extracellular domain of HER2 are effective in treating HER2-overexpressing BC, either alone or in combination with passive immunization. However, HER2 peptide-based vaccines, such as E75, and passive immunotherapies with HER2-targeting antibodies, like trastuzumab, have shown limited efficacy when used individually in tumors with low HER2 expression (IHC 1–2+) [281]. In a randomized phase IIb trial, trastuzumab was combined with E75 and granulocyte–macrophage colony-stimulating factor (GM-CSF) in 275 patients with low expressing HER2 BC at a high risk of recurrence. While the trial did not show a significant difference in disease-free survival (DFS) for the overall cohort, patients with TNBC who received E75 experienced improved DFS compared to the placebo group. TNBC, which lacks expression of ER, progesterone receptor (PgR), and HER2, tends to have higher immune infiltration and PD-L1 expression, making it potentially more responsive to immunotherapy. The findings suggest that the combination of trastuzumab’s immunologic effects, such as antibody-dependent cell-mediated cytotoxicity (ADCC) and cytotoxic T-cell stimulation, with HER2-targeted vaccines or checkpoint inhibitors could offer promising treatment improvements for TNBC [282] as well as other HER2-positive cancers. This highlights the potential for synergistic therapeutic strategies in these challenging cancer subtypes [281].

- Epitopes from HER2 transmembrane domain

GP2 is a 9-amino-acid peptide (IISAVVGIL) derived from the epitope spanning residues 654 to 662 of the HER2/neu protein, which is situated in its transmembrane region. This peptide is restricted by human leukocyte antigen (HLA)-A2 and is designed to stimulate cytotoxic T lymphocytes (CTLs) to recognize and destroy HER2/neu-expressing cancer cells [264,283]. Several preclinical studies have shown GP2 peptide-based vaccine efficacy, including in a mice xenograft model using HER2/neu-overexpressing tumors (e.g., TUBO cells), when it is incorporated into liposomes formulation [284] or displayed on Bacteriophage λ [285]. The formulation of liposomal GP2 elicited robust CD8+ T-cell responses, and both systems resulted in significantly smaller tumor sizes and longer survival times in both prophylactic and therapeutic settings compared to other treatment groups. In clinical trials, GP2 peptide vaccines proved to be safe and well-tolerated [286,287], increasing in circulating GP2-specific CD8+ T-cells, particularly in patients without pre-existing immunity to GP2 and an enhanced delayed type hypersensitivity (DTH) response to the GP2 peptide [286].

A single-blinded phase II clinical trial compared the effects of GP2+GM-CSF versus GM-CSF alone in high-risk, human leukocyte antigen (HLA)-A2/A3-positive BC patients who were disease-free following standard therapy. The primary outcome was disease recurrence. Patients were randomized to receive six monthly injections of GP2 with granulocyte–macrophage colony-stimulating factor (GM-CSF) or GM-CSF alone. Toxicity was monitored using the NCI toxicity scale, and immune responses were measured both in vivo via delayed-type hypersensitivity (DTH) reactions and in vitro through GP2-specific CTL assays. The results showed comparable clinicopathologic characteristics and toxicity profiles between the two groups. The GP2+GM-CSF group had a significantly larger immune response than the GM-CSF group. The percentage of GP2-specific CTLs significantly increased from baseline 6 months after completion of the inoculation series in the GP2+GM-CSF group. At a median follow-up of 17.9 months, recurrence rates were 7.4% in the GP2+GM-CSF group and 13% in the GM-CSF group. These early results, based on 50 of the planned 200 patients, suggest the trial is progressing well, with a final analysis planned after three years of follow-up [288].

In a randomized, blinded, controlled phase II trial, the GP2 peptide vaccine was applied in HLA-A2 positive, HER2/neu-expressing tumor (IHC 1–3+), disease-free node positive, and high-risk node negative BC patients enrolled after standard-of-care therapy. The primary objectives of this study were to determine if GP2 in combination with GM-CSF vaccination improves the DFS in any level of HER2 expression. The results showed no significant difference in disease-free survival (DFS) between the GP2 group and GM-CSF control group. However, GP2 improved clinical outcomes in the HER2 over-expressing subgroup, with no recurrences observed in the 5-year estimated DFS compared to the control [289].

The GP2 peptide vaccine may be beneficial in patients with HER2-overexpressing tumors who received trastuzumab as part of their standard-of-care treatment [290]. Trastuzumab increased the sensitivity of the tumor cells to CTL-mediated lysis after stimulation with either GP2 or E75, even in patients with low levels of HER2 expression [291]. Combined therapy of GP2 with other HER2 epitopes, including E75, have been promising in generating an additive effect [289]. The GP2+E75 stimulated CD8+ T-cells lysing HER2/neu+ targets in ex vivo [292].

- Epitopes from HER2 intracellular domain

The ICD of HER2, a tyrosine kinase domain comprising residues 712 to 1255, plays a critical role in the HER2/neu protein. Vaccines encoding the HER2 ICD have demonstrated both immunogenicity and protective effects against mammary tumors in pre-clinical models. In particular, a vaccine formulation combining the HER2 ICD with the heat shock complex HSP110 has been shown to elicit strong antitumor immune responses and effectively inhibit spontaneous mammary tumors in a rat neu transgenic mouse model [293]. In clinical studies, patients with HER2-positive BC who received a DNA-based vaccine encoding the HER2 ICD developed robust HER2-specific immunity [294]. A phase I/II trial investigated a peptide-based vaccine targeting the HER2 ICD, which included peptides p776–790 (GVGSPYVSRLLGICL), p927–941 (PAREIPDLLEKGERL), and p1166–1180 (TLERPKTLSPGKNGV). This vaccine was administered to patients with refractory HER2+ stage IV breast cancer, accompanied by leukapheresis for T-cell expansion, infusion with immunizing peptides, and booster injections. The trial results were promising, since 82% of patients showed an increase in HER2-specific T-cells in peripheral blood, targeting at least one of the immunizing epitopes, and 81% exhibited intramolecular epitope spreading. Importantly, the treatment was associated with improved overall survival in the responding patients [212].

HER2-negative tumors can sometimes express the HER2 ICD, displaying a HER2-positive phenotype. Current HER2 testing primarily assesses the expression of the full HER2 protein on the cell surface, potentially overlooking cases where only the intracellular domain is expressed. A tumor with a genetic mutation may produce a truncated HER2 protein that lacks the ECD but retains the active signaling ICD. If a HER2-negative tumor expresses the HER2 ICD, it may still partially respond to HER2-targeted therapies, underscoring the need for further investigation into such cases [295].

The AE37 epitope is a modified form of the native HER2 protein fragment, specifically derived from the intracellular domain of HER2 (aa 776–790). This region corresponds to the naturally occurring HER2 peptide, AE36 (776–790, G89), but AE37 includes a key modification—a 4-amino-acid extension known as the Ii-Key modification (LRMK) added to the N-terminus [296]. The purpose of the Ii-Key modification is to enhance the presentation of the AE37 peptide to MHC class II molecules, thereby improving its ability to activate CD4+ helper T-cells. The enhanced CD4+ T-cell response improves immune system coordination, leading to stronger cytotoxic CD8+ T-cell responses and a more effective overall antitumor immune response.

Although AE37 is primarily designed to target CD4+ T-cells, studies have demonstrated its ability to also activate CD8+ T-cells, resulting in a more comprehensive and potent immune response against HER2-expressing cancer cells. Moreover, the immune response triggered by AE37 has the capacity to recognize and respond to related peptides, such as AE36, another HER2-derived peptide, potentially broadening the overall antitumor response. This dual activation of CD4+ and CD8+ T-cells in response to HER2-derived peptides underscores AE37’s potential as a therapeutic vaccine candidate, particularly for HER2-positive cancers, including breast cancer [297].

In a randomized, blinded, controlled phase II trial, the AE37 peptide vaccine was applied in HLA-A2 negative, HER2/neu-expressing tumor (IHC 1–3+), disease-free node positive, and high-risk node negative breast cancer patients enrolled after standard-of-care therapy. The primary objectives of this study were to determine if AE37 in combination with GM-CSF vaccination improves the DFS in any level of HER2 expression. The results showed no significant difference in disease-free survival (DFS) between the AE37 group and GM-CSF control group. However, AE37 improved clinical outcomes in the advanced stage, HER2 under-expression, and TNBC subgroup, with no recurrences observed in the 5-year estimated DFS compared to the control [289].

#### 4.5.2. MUC1

Another peptide-based vaccine being explored for breast cancer targets the transmembrane glycoprotein MUC1. While MUC1 is typically present on the apical surface of epithelial cells, it is often overexpressed in cancer cells, with levels up to 100 times higher than those found in normal cells. Moreover, the glycosylation patterns of MUC1 in cancer cells are frequently abnormal. Research has demonstrated that the production of antibodies against MUC1 and the activation of cellular immune responses can positively influence cancer patient outcomes. Consequently, enhancing MUC1-specific immunity through approaches such as vaccines, MUC1-targeted antibodies, or T-cell therapies is a promising strategy in cancer treatment [298].

A vaccine targeting MUC1 can stimulate a tumor-specific immune response, but this effect is often suppressed within the breast tumor microenvironment. Studies have shown that cyclooxygenase-2 (COX-2) is frequently overexpressed in breast carcinoma, and its elevated levels are associated with poor prognosis [299]. COX-2 overexpression impairs the function of cytotoxic CD8+ T lymphocytes, T helper (Th) lymphocytes, natural killer (NK) cells, and dendritic cells (DCs) [300], thereby diminishing the effectiveness of anti-breast cancer vaccines.

Curry et al. (2019) investigated the effectiveness of MUC1 vaccination in combination with four different drugs that inhibit different components of the COX pathway: indomethacin (COX-1 and COX-2 inhibitor), celecoxib (COX-2 inhibitor), 1-methyl tryptophan (indoleamine 2,3 dioxygenase inhibitor), and AH6809 (prostaglandin E2 receptor antagonist). These treatment regimens were explored for the treatment of orthotopic MUC1-expressing breast tumors in mice transgenic for human MUC1. The vaccine formulation included two MHC class I-restricted MUC1 peptides, APGSTAPPA and SAPDTRPAP; an MHC class II helper peptide, TPPAYRPPNAPIL (derived from the Hepatitis B virus core antigen sequence 128–140); mouse unmethylated CpG oligodeoxynucleotide constructs (CpG ODN); and GM-CSF, all emulsified in IFA. This vaccine was administered to female mice both with and without a COX-2 inhibitor at various time points, following orthotopic tumor cell injection into the mammary fat pad. The results demonstrated that both the MUC1 peptide vaccine and the combination therapy led to elevated levels of anti-MUC1-specific IFN-γ-producing T-cells and antibody responses in vivo. Notably, while indomethacin significantly enhanced the efficacy of the MUC1 peptide vaccine, it did not improve the vaccine’s antitumor activity. In contrast, the combination therapy increased the number of cancer cells undergoing apoptosis, resulting in a substantial reduction in tumor burden [265].

#### 4.5.3. Multi-Peptide Vaccines

Additionally, multi-peptide vaccines for breast cancer represent a promising approach. An early phase II study evaluated a vaccine composed of 19 distinct peptides derived from 11 different TAAs known to induce cytotoxic T lymphocytes (CTLs). These TAAs included SART3, CypB, WHSC2, UBE2V, HNRPL, Lck, MRP3, PSA, PAP, EGFR, and PTHrP. Fourteen patients with advanced metastatic TNBC, who had not responded to chemotherapy, received this vaccine, which was emulsified with incomplete Freund’s adjuvant and administered subcutaneously in the abdominal region on days 1, 8, 15, 22, 29, and 36. The study’s primary endpoint was safety, while secondary endpoints included the specific immune response against the selected peptides and overall survival.

A significant increase in peptide-specific IgG antibodies was observed post-vaccination, with IgG levels reaching up to 74,943 FIU, compared to pre-vaccination levels of 53–14,482 FIU, particularly in 9 of the 10 patients who completed all six vaccinations, correlating with prolonged overall survival (OS). Patients demonstrating high IgG titers had a median OS of 24.0 months, significantly longer than those in previous anti-PD-1/PD-L1 trials, with responses to Lck-486, PAP-213, CypB-129, PSA-248, and UBE2V-43 correlating with statistically significant survival benefits, emphasizing their potential role in durable immune responses and antitumor effects.

CTL responses, assessed via IFNγ release, were robust in 5 of the 10 patients, though initially suppressed, reflecting tumor-specific cellular immunity targeting HLA-matched peptides; whereas, responses to CEF peptide pools remained unchanged, reinforcing vaccine specificity. Notably, CTL activity demonstrated lower sensitivity for immune boosting than IgG responses, yet their co-occurrence suggested a synergistic effect, supporting comprehensive immune activation against tumors. Variations in CTL responses were attributed to immune suppression due to prior chemotherapy, with higher baseline C-reactive protein (CRP) levels and extensive chemotherapy regimens reducing CTL boosting, highlighting the influence of pretreatment factors on vaccine efficacy.

Expression analysis of 11 TAAs in tumor samples confirmed that HNRPL, WHSC2, SART3, CypB, PTHrP, and UBE2V were expressed in most tumor cells, validating their immunogenicity, while pre-existing IgG responses in patient plasma further supported their potential for immune enhancement. The phase II clinical trial demonstrated significant immune boosting in most patients following vaccination, with heightened IgG and CTL responses suggesting prolonged immunity and tumor protection. The association between immune responses and OS, particularly with Lck, PAP, CypB, PSA, and UBE2V peptides, underscores their therapeutic relevance, indicating that this mixed 19-peptide vaccine is a promising immunotherapy for advanced cancer [270].

Despite the small sample size limiting statistical power, these findings warrant further investigation in larger cohorts to validate the vaccine’s potential for clinical application.

#### 4.5.4. MAGEA

The MAGEA gene family, or melanoma antigen family A is a group of genes that encode proteins involved in cancer. MAGEA proteins are expressed in reproductive tissues but are abnormally expressed in many types of cancer, including melanoma, pancreatic cancer, breast cancer, and ovarian carcinoma. MAGEA proteins are associated with poor patient survival, invasion, and metastasis.

Twelve vaccine formulations designed with a MAGE-A1 antigenic peptide (SQYGEPRKL) conjugated to antigenic peptide-TLR2 agonist and self-assembled into nanoparticles were tested in pre-clinical study. All the conjugate formulations showed efficient take up by DC and induced DC maturation, activated CD8+ T-cells, and showed cytotoxicity against MAGE-A1 positive cancer cells MCF-7 in vitro. However, among the different conjugated formulations, the one with conjugate 6 (N-Ac PamCS-M-6) showed better antitumor efficacy and safety in MCF-7 tumor-bearing BALB/c nude mice [267].

Several epitopes (e.g., MLP1-3 and MLP-A4) derived from MAGE-A4 were utilized in a multi-epitope vaccine during pre-clinical studies in Balb/c mice. These mice were inoculated subcutaneously with either 4T1-MAGE-A4+ or 4T1-MAGE-A4− cells. The peptide mixtures successfully induced an immune response, generating multi-epitope-specific cytotoxic T lymphocytes (CTLs) restricted to the HLA-A1, HLA-A2, HLA-A3, and HLA-A24 superfamily. The results demonstrated that the multi-epitope vaccine, both alone and in combination with R848, an epitope associated with cancer, exhibited significant antitumor activity [268].

#### 4.5.5. Triple Peptide Vaccination

In addition to MUC1 and HER2 (ErbB2), CEA is also overexpressed in both BC and ovarian cancer (OC). A triple peptide vaccine composed of HLA-A2-restricted peptides, MUC1 (159–167), ErbB2 (368–377), or CEA (605–613) was evaluated in a phase I/II clinical trial. The trial enrolled patients with a median age of 53, all of whom had high-risk, disease-free BC or OC. These patients had completed the standard-of-care treatment appropriate for their tumor type and stage and were HLA-A2 positive. Prior to vaccination, none of the patients exhibited a specific CD8+ IFNγ-producing immune response. Vaccination commenced within six months of completing chemotherapy. The treatment regimen consisted of six consecutive doses administered biweekly, followed by a booster dose three months after the final dose. The vaccine was found to be safe and well-tolerated. Out of the 14 patients enrolled, 8 developed CD8+ T-cell responses specific to at least one of the targeted antigens. For survival analysis, patients were followed for eight years post-treatment. At the eight-year follow-up, all BC and OC patients remained alive and disease-free. Although three patients experienced disease recurrence, they were successfully treated with surgery and adjuvant chemotherapy [266].

### 4.6. Advantages and Disadvantages of Anticancer Vaccines in Breast Cancer Treatment

Anticancer vaccines are an emerging strategy in breast cancer therapy, aiming to stimulate the immune system to recognize and attack cancer cells. These vaccines can be preventive (prophylactic) or therapeutic (treatment-based) and are designed to target tumor-associated antigens (TAAs) or neoantigens.

#### 4.6.1. Advantages of Anticancer Vaccines for Breast Cancer

- Specific immune activation: anticancer vaccines stimulate the immune system to recognize and eliminate cancer cells, minimizing damage to normal tissues compared to chemotherapy or radiation [301].
- Potential for long-term protection: once trained, the immune system can maintain memory against cancer antigens, reducing the risk of recurrence and metastasis [302].
- Low toxicity compared to traditional therapies: unlike chemotherapy and radiation, vaccines generally have fewer systemic side effects, since they do not directly kill cells but rather enhance immune surveillance [303].
- Potential to target minimal residual disease: vaccines may help eliminate residual cancer cells after surgery, reducing the likelihood of relapse [304].
- Application in high-risk patients: individuals with high genetic risk (e.g., BRCA1/2 mutations) could benefit from prophylactic cancer vaccines in the future, similar to HPV vaccines for cervical cancer prevention [305].
- Combination potential with other therapies: cancer vaccines can be combined with immune checkpoint inhibitors (e.g., anti-PD-1/PD-L1), chemotherapy, or radiotherapy to enhance therapeutic efficacy [306].

#### 4.6.2. Disadvantages of Anticancer Vaccines for Breast Cancer

- Limited efficacy in established tumors: breast tumors often create an immunosuppressive microenvironment, making it difficult for vaccines to generate a strong immune response [307].
- Tumor antigen heterogeneity: breast cancer is highly heterogeneous, and different patients may express different tumor antigens, making it challenging to develop a universal vaccine [308].
- Slow onset of action: unlike chemotherapy or targeted therapy, which can show effects quickly, vaccines take time to stimulate an immune response, making them less effective for patients with rapidly progressing disease.
- Risk of autoimmune reactions: some tumor-associated antigens are also present in normal tissues, and immune activation against these antigens may cause autoimmune side effects [309].
- Need for effective biomarker identification: predicting which patients will respond to cancer vaccines is challenging, requiring the identification of biomarkers to select appropriate candidates [310].
- Limited success in clinical trials: while promising in preclinical studies, many anticancer vaccines for breast cancer have failed in clinical trials due to weak immunogenicity or lack of efficacy in advanced disease.
- High cost and regulatory challenges: developing personalized or peptide-based cancer vaccines is expensive, and regulatory approval can be slow due to the need for extensive clinical validation.

In conclusion, anticancer vaccines hold promise as a future approach for breast cancer prevention and treatment, particularly in early-stage disease or high-risk populations. However, challenges such as tumor immune evasion, antigen selection, and clinical efficacy must be addressed through improved vaccine designs, combination therapies, and biomarker-driven patient selection.

## 5. Future Directions

**Figure 2:**
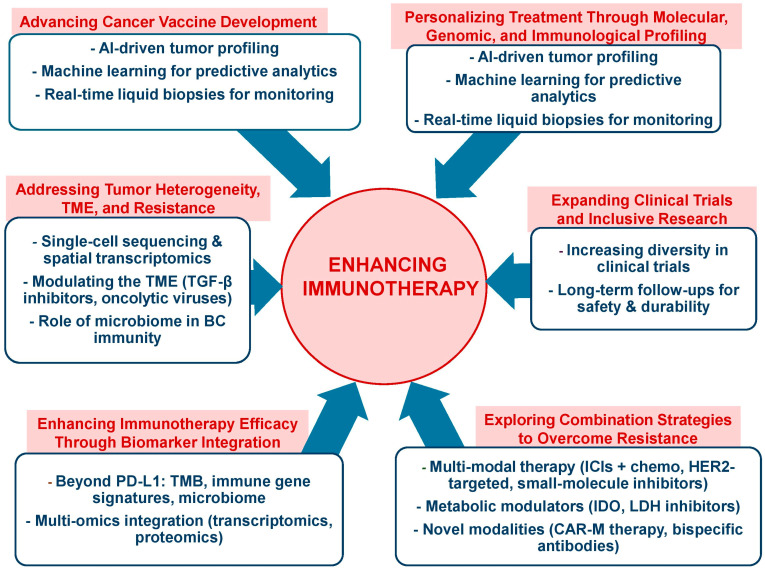
Future research directions in breast cancer immunotherapy.

Breast cancer remains a major global health challenge due to its biological complexity and heterogeneity, which significantly impact treatment efficacy. While advancements in targeted therapies and immunotherapy have improved patient outcomes, several critical areas require further research to optimize treatment strategies, enhance efficacy, and overcome existing limitations (Figure 2).

### 5.1. Enhancing Immunotherapy Efficacy Through Biomarker Integration

Despite the success of immune checkpoint inhibitors (ICIs) in some BC subtypes, patient responses remain highly variable. Future research should focus on identifying reliable predictive biomarkers beyond PD-L1 expression, such as tumor mutation burden (TMB), immune gene signatures, and gut microbiome composition. Integrating multi-omics approaches, including transcriptomics and proteomics, will provide deeper insights into tumor-immune interactions and help stratify patients for personalized immunotherapy.

### 5.2. Addressing Tumor Heterogeneity, TME, and Resistance Mechanisms

Tumor heterogeneity poses a significant challenge in BC treatment, leading to immune escape and therapy resistance. Future studies should explore the clonal evolution of BC cells and their interactions with the TME to develop adaptive therapeutic strategies. Single-cell sequencing [311] and spatial transcriptomics [312] can be leveraged to dissect intratumoral heterogeneity, while functional studies should focus on the mechanisms of resistance to ICIs and cancer vaccines.

The TME plays a crucial role in BC progression and treatment resistance. Understanding the interactions between immune cells, stromal components, and tumor cells will facilitate the development of novel strategies to modulate the TME. Combining immunotherapy with agents that modulate the TME, such as TGF-β inhibitors and oncolytic viruses [313], could enhance treatment efficacy and durability.

Research should explore immune-modulating agents that can enhance antigen presentation, promote T-cell infiltration, and counteract immunosuppressive factors within the TME. Additionally, investigating how microbiome composition influences BC immunity may offer novel therapeutic avenues.

### 5.3. Advancing Cancer Vaccine Development

Epitope-based cancer vaccines targeting TAAs, such as HER2, MUC1, CEA, and MAGE-A, have shown promise in preclinical and early clinical trials. However, their efficacy remains limited due to immune tolerance and weak immunogenicity. Future research should focus on optimizing antigen selection, incorporating neoantigens, and improving vaccine delivery methods, such as nanoparticle-based platforms or dendritic cell vaccines. Additionally, combining cancer vaccines with ICIs or adoptive cell therapies may enhance immune activation and improve clinical outcomes.

### 5.4. Personalizing Treatment Through Molecular, Genomic, and Immunological Profiling

A more personalized approach to BC treatment is crucial for maximizing therapeutic success. Future research should prioritize the integration of molecular, genomic, and immunological profiling to refine patient selection for immunotherapy. Advances in single-cell sequencing, transcriptomics, and proteomics will allow for more precise patient stratification, leading to better therapeutic targeting.

The development of artificial intelligence (AI)-driven models for analyzing patient-specific tumor and immune characteristics will aid in predicting responses to different therapeutic modalities [314]. Machine learning tools can be harnessed to analyze complex datasets, predict treatment responses, and identify novel therapeutic targets. Furthermore, real-time liquid biopsy techniques should be expanded to monitor treatment response and detect resistance at an early stage [315].

### 5.5. Exploring Combination Strategies to Overcome Resistance

Overcoming drug resistance remains a major challenge in BC therapy. Future research should focus on elucidating the mechanisms underlying resistance to hormone therapy, targeted therapy, and immunotherapy. Identifying novel druggable targets and developing combination strategies that address resistance pathways will be key to enhancing long-term treatment efficacy. Potential avenues include combining ICIs with chemotherapy, HER2-targeted therapies, or small-molecule inhibitors targeting immunosuppressive pathways. The exploration of metabolic modulators, such as IDO inhibitors [316] and lactate dehydrogenase (LDH) inhibitors [317], may also help reprogram the immunosuppressive TME, making tumors more susceptible to immune attack. Integrating novel therapeutic modalities, such as CAR-M therapy or bispecific antibodies, could revolutionize BC management.

### 5.6. Expanding Clinical Trials and Inclusive Research

Current clinical trials often have limited patient diversity, which can hinder the generalizability of findings. Future research efforts should prioritize expanding clinical trial participation among under-represented populations to ensure the broad applicability of novel therapies. Additionally, long-term follow-up studies are necessary to assess the durability of immunotherapy responses and identify potential late-onset toxicities.

In conclusion, advancing BC treatment requires a multidisciplinary approach, integrating molecular profiling, innovative immunotherapeutic strategies, the integration of cutting-edge technologies, and personalized treatment regimens. By addressing tumor heterogeneity, refining biomarker-driven patient selection, and exploring synergistic treatment combinations, future research can significantly improve patient outcomes and pave the way for more effective and durable BC therapies.

## 6. Discussion

Breast cancer remains a significant global health challenge due to its heterogeneity, which influences treatment response and therapeutic success. The advent of immunotherapy and cancer vaccines has introduced promising alternatives to conventional treatments, aiming to leverage the immune system to selectively target cancer cells, while minimizing toxicity. However, the effectiveness of these therapies is highly dependent on tumor-specific factors, such as breast cancer subtype, TMB, TILs, PD-L1 expression, HER2 resistance, and the TME. Understanding these factors is critical for improving patient stratification and optimizing therapeutic efficacy.

Among BC subtypes, TNBC has demonstrated the highest responsiveness to immunotherapy due to its higher immunogenicity and greater prevalence of immune-infiltrating cells. In contrast, HER2+ and HR+ breast cancers exhibit lower innate immunogenicity, requiring combination strategies that integrate ICIs with HER2-targeted therapies or endocrine treatments. High TMB correlates with improved response rates to ICIs by increasing neoantigen presentation; whereas, low TMB is associated with immune evasion. Similarly, PD-L1 expression serves as a predictive biomarker for checkpoint inhibitor therapy, with higher expression levels correlating with better responses. The TME, particularly in HR+ and HER2+ breast cancers, often features an immunosuppressive landscape, which contributes to therapeutic resistance and necessitates innovative strategies to reprogram the immune milieu.

Current immunotherapies for breast cancer encompass a broad spectrum of modalities, including monoclonal antibodies (trastuzumab, pertuzumab), small-molecule inhibitors (lapatinib, tucatinib), ADCs, and ICIS (pembrolizumab, atezolizumab). While ICIs have demonstrated clinical success, their efficacy remains limited in HR+ and HER2+ subtypes, necessitating combination approaches with chemotherapy, targeted therapies, or novel immune-modulating agents. Additionally, CAR-M therapy represents a pioneering strategy to remodel the TME and enhance antitumor immune responses, offering new avenues for overcoming immunotherapy resistance.

Cancer vaccines, particularly mRNA, dendritic cell, and peptide-based formulations, have gained attention for their ability to prime immune responses against tumor-associated antigens. While these vaccines hold promise, clinical outcomes have been inconsistent due to tumor antigen heterogeneity, weak immune activation in advanced disease, and immune escape mechanisms. Overcoming these challenges requires improved antigen selection, enhanced adjuvant formulations, and combination strategies that synergize with ICIs or chemotherapy. Ongoing research aims to refine vaccine efficacy through biomarker-driven approaches, optimizing immunogenicity and patient selection criteria.

Despite the transformative potential of immunotherapy and cancer vaccines, several challenges remain. Immune-related adverse events (irAEs), high costs, and delayed treatment responses (e.g., pseudo-progression) present significant barriers to widespread clinical adoption. Resistance mechanisms, including immune escape, TME-mediated suppression, and the upregulation of alternative immune checkpoints, necessitate continuous innovation in combination therapies and predictive biomarker identification. Additionally, patient heterogeneity underscores the need for personalized treatment approaches integrating genomic, proteomic, and immunological profiling.

## 7. Conclusions

Immunotherapy and cancer vaccines have revolutionized the landscape of breast cancer treatment, offering novel strategies to harness the immune system against malignancy. While checkpoint inhibitors and immune-modulating therapies have demonstrated success, their efficacy remains variable across breast cancer subtypes, necessitating tailored approaches based on predictive biomarkers. Cancer vaccines, although promising, require further optimization to improve clinical efficacy, particularly in combination with existing immunotherapies.

Future research must focus on overcoming resistance mechanisms, refining patient stratification through biomarker-driven approaches and developing next-generation therapies that remodel the TME to enhance immune responsiveness. Advances in single-cell sequencing, AI-driven predictive models, and novel immune-modulating agents will be crucial in addressing tumor heterogeneity and resistance pathways. Additionally, expanding clinical trials with diverse patient populations and long-term follow-up will be essential for translating emerging therapies into widespread clinical use.

Ultimately, a multidisciplinary approach integrating immunotherapy, cancer vaccines, and personalized medicine holds the key to improving breast cancer treatment outcomes. As research progresses, innovative combination strategies and targeted immune interventions will play a pivotal role in achieving durable remissions and enhancing survival rates for breast cancer patients.

## Figures and Tables

**Table 1:** Main factors that affect breast cancer immunotherapy decisions.

Factors	Description	Reference
BC type	By determining immune landscape and treatment response. TNBC responds best to immunotherapy, while HER2+ and HR+ subtypes may require combination approaches for enhanced efficacy.	[5]
TMB	By influencing neoantigen production and immune activation. High TMB enhances response to immune checkpoint inhibitors, while low TMB may indicate resistance to immunotherapy.	[6]
TILs	By reflecting antitumor immune activity. High TIL levels correlate with better response to immune checkpoint inhibitors, while low TILs may indicate immune evasion and reduced efficacy.	[7]
PD-L1 expression	By predicting response to immune checkpoint inhibitors. High PD-L1 levels enhance immunotherapy efficacy, while low expression may indicate limited benefit from checkpoint blockade therapies.	[8]
HER2 resistance	By limiting response to HER2-targeted therapies and altering tumor immune evasion. Overcoming resistance may require combination strategies, including immune checkpoint inhibitors and novel immunotherapies.	[9]
TME	By comprising cancer cells, stromal components, blood vessels, and immune cells, all of which dynamically interact to influence tumor progression and therapy response.	[10]

BC, breast cancer; TNBC, triple-negative breast cancer; TMB, tumor mutation burden; TILs, tumor-infiltrating lymphocytes; PD-L1, programmed cell death ligand 1; HER2, human epidermal growth factor receptor 2; TME, tumor microenvironment.

**Table 2:** Factors influencing immunotherapy outcomes across various studies.

Factor	Type BC	Value	Luminal A (HR+, HER2−)	Luminal B (HR+, HER2+)	HER2-Enriched (HR−, HER2+)	TNBC (HR−, HER2−)	Ductal	Lobular	Ref.
TMB threshold ≥ 10 mut/MB	Metastatic BC	%High (total number)	7.8% (3087)	8.3% (266)	7.8% (179)	8.6% (1476)	5.1% (2273)	16% (349)	[11,12]
TIL	Early breast cancer	%Low/%High (total number)	83%/17% (46)		45%/55% (42)	27%/73% (92)			[13]
	Invasive breast cancers	%TIL density (total number)	11.1% (162)		31.7% (101)	38.1% (147)			[14]
PD-1 Expression	Invasive breast tumor	Percent (total number)	40% (10)	47.8% (23)	60% (10)	50% (4)			[15]
	Metastatic lymph node		20% (10)	14%	5%	75%			[15]
	Early breast cancer		13% (46)		19% (42)	28% (92)			[13]
PD-L1 Expression	Invasive breast cancers	Percent (total number)	53.1% (162)		73.3% (101)	84.4% (147)			[14]
	Invasive breast tumor		20% (10)	34.7% (23)	20% (10)	50% (4)			[15]
	Metastatic lymph node		0% (10)	17.4% (23)	10% (10)	50% (4)			[15]
	Early breast cancer		20% (46)		31% (42)	43% (92)			[13]
TME	Metastatic BC	Median T-cell -imflamed score	3.96	4.02	4.2	4.17	4.14	3.90	[11]

TMB, tumor mutation burden; TIL, tumor-infiltrating lymphocytes; TME, tumor microenvironment.

**Table 3:** Main breast cancer immunotherapies.

Therapy/Drug	Description	Reference
HER2 Block		
Monoclonal Antibodies	Trastuzumab binds to subdomain IV, blocking HER2 dimerization and activating antibody-dependent cellular cytotoxicity (ADCC). It is a key treatment for HER2-positive breast and gastric cancers, significantly improving survival. Pertuzumab targets subdomain II, preventing HER2/HER3 dimerization and enhancing trastuzumab’s effects when combined with chemotherapy. This combination is a standard treatment for HER2-positive metastatic breast cancer, offering superior outcomes.	[79]
Small Molecule TKIs	Lapatinib and tucatinib inhibit HER2’s intracellular kinase domain, blocking tumor growth. Tucatinib is FDA-approved for combination therapy, while lapatinib helps overcome trastuzumab resistance in HER2-positive breast cancer.	[80]
ADCs	ADCs combine monoclonal antibodies with cytotoxic agents for targeted cancer therapy. They selectively bind tumor antigens, internalize, and release potent drugs, minimizing toxicity. ADCs offer precision treatment, bystander effects, and immune activation, enhancing efficacy in resistant cancers.	[81]
Immune Checkpoint Inhibition		
PD-1/PD-L1	PD-1, an inhibitory receptor on T-cells, suppresses antitumor immunity via interactions with PD-L1/PD-L2. Checkpoint inhibitors, like pembrolizumab and atezolizumab, block this pathway, restoring T-cell function and enhancing cancer immunotherapy efficacy.	[82]
CTLA-4	CTLA-4 inhibits T-cell activation by outcompeting CD28 for B7 ligands on APCs. Ipilimumab blocks CTLA-4, restoring CD28 signaling and enhancing T-cell activation, leading to stronger antitumor immune responses in cancer immunotherapy.	[83]
CAR-M	CAR-M harness macrophages’ phagocytic and antigen-presenting abilities to target tumors and reshape the immunosuppressive tumor microenvironment. CAR-M therapy enhances immune activation, overcomes resistance to immunotherapy, and shows promise in breast cancer treatment.	[84]

TKIs, tyrosine kinase inhibitors; ADCs, antibody–drug conjugates; PD-1, programmed cell death protein 1; PD-L1, programmed cell death ligand 1; CTLA-4, cytotoxic T-lymphocyte-associated antigen 4; CAR-M, chimeric antigen receptor macrophages.

**Table 4:** Breast cancer vaccines.

Therapy/Drug	Description	Reference
Protein- or Whole-Cell-Based Vaccines	Protein-based vaccines, like HER2 vaccines, enhance antibody and T-cell activation, while whole-cell vaccines provide broad antigen coverage, reducing immune escape. These vaccines hold promise for cancer immunotherapy, especially in combination therapies.	[204]
DNA and RNA Vaccines	DNA and RNA vaccines use genetic material to stimulate immune responses against tumor-associated antigens. DNA vaccines rely on plasmid DNA to encode tumor antigens, while mRNA vaccines provide direct instructions for antigen production. mRNA vaccines have shown promise, particularly for HER2-positive cancers, due to their rapid development and strong immune activation.	[205]
Dendritic Cell (DC) Vaccines	Dendritic cell (DC) vaccines utilize the antigen-presenting abilities of dendritic cells to stimulate targeted anticancer immune responses. These vaccines are created by isolating a patient’s dendritic cells, exposing them to tumor antigens, and reintroducing them to activate T-cells against cancer.	[206]
Viral Vector-Based Vaccines	Viral vector-based vaccines use modified viruses, such as adenoviruses or poxviruses, to deliver tumor-associated antigen (TAA) genes into host cells, stimulating both humoral and cellular immune responses against cancer.	[207]
Epitope-Based Vaccines	Peptide-based vaccines target tumor-associated (TAAs) and tumor-specific antigens (TSAs) to stimulate immune responses, while minimizing damage to healthy tissues.	[208]

**Table 5:** Sequences from different epitopes used in epitopes-based vaccines against breast cancer.

Name	Origin Protein	Position	Amino Acid Sequence	Reference
P5	HER2/neu	ECD	ELAAWCRWGFLLALLPPGIAG	[258]
P435	HER2	ECD	IRGRILHDGAYSLTLQGLGIH	[258]
P5+435	HER2	ECD	ELAAWCRWGFLLALLPPGIAGRRIRGRILHDGAYSLTLQGLGIHGGGC	[258]
CH401	HER2	163–182	YQDMVLWKDVFRKNNQLAPV	[259]
E75 or NPS or ErbB2_368–377_	HER2	366–379	KIFGSLAFL	[260]
E90	HER2	ECD	CLTSTVQLV	[260]
P467	HER2	ECD	PESFDGDPASNTAPLQPRVLQGLPREYVNARHSLPYMPIWKFPDEEGAC	[261]
JTMP	HER2/neu	260–301	HSGICELHCPALVTYNTDTFESMPNPEGRYTFGASCVTACPY	[262]
P101	HER2	ECD	RLRIVRGQLFEDKYAL	[263]
P373	HER2	ECD	KIFGSLAFLPESFDGDPS	[263]
GP2	HER2	654–662	IISAVVGIL	[264]
AE36, AE37, p776–790	HER2	776–790	GVGSPYVSRLLGICL	[212]
p927–941	HER2	927–941	PAREIPDLLEKGERL	
p1166–1180	HER2	166–1180	TLERPKTLSPGKNGV	
MUC1_159–167_	MUC1	159–167	SAPDNRPAL	[265]
CEA_605–613_	CEA	605–613	YLSGADLNL	[266]
MAGE-A1	MAGEA		SQYGEPRKL	[267]
MLP1	MAGE-A4	102−130	AESLFREALSNKVDELAHFLLRKYRAKEL	[268]
MLP2	MAGE-A4	134−160	AEMLERVIKNYKRCFPVIFGKASESLK	[268]
MLP3	MAGE-A4	274−306	RALAETSYVKVLEHVVRVNARVRIAYPSLR	[268]
SART3				
SART3-302	SART3	302–310	LLQAEAPRL	[269]
SART3-309	SART3	309–317	RLAEYQAYI	[269]
SART3-109	SART3	109–118	VYDYNCHVDL	[269]
SART3-734	SART3	734–742	QIRPIFSNR	[269]
CypB-129	Cyclophilin B	129–138	KLKHYGPGWV	[269]
WHSC2				
WHSC2-103	WHSC2	103–111	ASLDSDPWV	[269]
WHSC2-141	WHSC2	141–149	ILGELREKV	
UBE2V-43	UBE2V	43–51	RLQEWCSVI	[269]
HNRPL-140	HNRPL	140–148	ALVEFEDVL	[269]
Lck				
Lck-246	p56^lck^	246–254	KLVERLGAA	[269]
Lck-208	p56^lck^	208–216	HYTNASDGL	[269]
Lck-486	p56^lck^	486–494	TFDYLRSVL	[269]
Lck-488	p56^lck^	488–497	DYLRSVLEDF	[269]
Lck-90	p56^lck^	90–99	ILEQSGEWWK	[269]
Lck-449	p56^lck^	449–458	VIQNLERGYR	[269]
MRP3-1293	MRP3	1293–1302	NYSVRYRPGL	
PSA-248	PSA	248–257	HYRKWIKDTI	[270]
PAP-213	PAP	213–221	LYCESVHNF	[270]
EGFR-800	EGFR	800–809	DYVREHKDNI	[270]
PTHrP-102	PTHrP	102–111	RYLTQETNKV	[270]

A3sup = HLA-A3 supertypes (A3, A11, A31, and A33). HLA = human leukocyte antigen. CypB, cyclophilin B; EGFR, epidermal growth factor-receptor; HNRPL, heterogeneous nuclear ribonucleoprotein L; Lck, p56lck; MRP3, multidrug resistance-associated protein 3; PAP, prostatic acid phosphatase; PSA, prostate-specific antigen; PSMA, prostate-specific membrane antigen; PTHrP, parathyroid hormone-related peptide; SART2, squamous cell carcinoma antigen 2; SART3, squamous cell carcinoma antigen 3; UBE2V, ubiquitin-conjugated enzyme variant Kua; WHSC2, Wolf–Hirshhorn syndrome critical region 2.

## Data Availability

All data used can be found in the text and tables.
